# Microbial metabolism affects the antibiotic resistome in the intestine of laying hens

**DOI:** 10.1016/j.psj.2024.104138

**Published:** 2024-07-29

**Authors:** Yilin Yuan, Chunhao Mo, Feng Huang, Xindi Liao, Yiwen Yang

**Affiliations:** ⁎ Guangdong Provincial Key Laboratory of Agro-Animal Genomics and Molecular Breeding, State Key Laboratory of Swine and Poultry Breeding Industry, College of Animal Science, South China Agricultural University, Guangzhou 510640, China; † School of Biological Engineering, Henan University of Technology, Zhengzhou 450001, China

**Keywords:** laying hen, intestine, microbial metabolism, antibiotic resistome

## Abstract

Intestinal microbial metabolism has an important impact on the health of laying hens, and microbes are also important hosts for ARGs. However, the relationship between intestinal microbes and antibiotic resistance in laying hens is unclear. In this study, a slaughtering experiment, an in vitro fermentation experiment and a single-bacteria culture experiment were carried out, and metagenomic and metabolomic analyses were used to investigate the relationships between microbial metabolism and the antibiotic resistome in the cecum of laying hens. The results showed that there were different types of ARGs in the intestines of laying hens, and the risk scores of the ARGs tended to decrease with growth stage. A total of 1142 metagenome-assembled genomes (**MAGs**) were obtained, and *Escherichia coli* was found to be the dominant ARG host, carrying 62 ARGs. Metabolomics revealed that indole and its derivatives, such as indole-3-lactic acid, were negatively correlated with a variety of ARGs. Moreover, in vitro fermentation experiment and single-bacteria culture experiment demonstrated that indole-3-lactic acid reduced the abundance and risk of multiple ARGs in the intestine and inhibited the growth of the ARG host *Escherichia coli*. In the context of high concern about intestinal microbial metabolism and antibiotic resistance, this is the first study to focus on the relationship between intestinal microbial metabolism and antibiotic resistance in laying hens. These findings have important implications for healthy farming and antibiotic resistance control.

## INTRODUCTION

Intestinal microorganisms play an important role in maintaining the integrity of the intestinal barrier, promoting the digestion and absorption of nutrients and other physiological activities. However, with the development of intensive farming, the use of antibiotics continues to increase in the production process of laying hens. Antibiotics induce the emergence of antibiotic-resistant bacteria and the enrichment of ARGs in the intestine, including tetracycline, aminoglycoside, and multidrug resistance genes (Hastings et al., 2004), triggering the problem of antibiotic-resistant contamination and affecting the healthy breeding of laying hens.

Studies have shown that the chicken intestine contains a high number of antibiotic-resistant bacteria. The main ARG hosts were *Escherichia, Enterococcus, Staphylococcus, Klebsiella, and Lactobacillus* (Yang et al., 2022). Among them, *Escherichia coli* often exhibits multidrug resistance and is also recognized as a major public health threat (Yang et al., 2014). These resistant bacteria not only lead to broader contamination through fecal elimination but also contribute to the spread of complex antibiotic-resistant contamination by transferring ARGs between different genera (Huang et al., 2022). Moreover, even after the antibiotic selection pressure has been removed, these resistant bacteria continue to exist in the environment, thereby causing long-lasting contamination effects. Therefore, it is crucial to prioritize addressing the risk of antibiotic resistance in the intestines of laying hens (Morrow, 2024).

Animal intestinal microorganisms perform metabolic activities by utilizing nutrients in the host intestine, and their metabolic processes have a positive effect on the host's nutrition and immunity (Mattila et al., 2018; Li et al., 2024). Normal metabolism of intestinal microorganisms ensures both the normal physiological activity of the microorganisms and the health of the host. However, it has been shown that bacterial metabolism can modulate antibiotic resistance. For example, metabolic modulation can turn resistant bacteria back to antibiotic-susceptible bacteria (Martínez and Rojo, 2011). However, the time period is relatively short, depending on the metabolic state of the bacterial population. It has also been shown that carbon sources and cellular respiration affect bacterial susceptibility to antibiotics (Vitko et al., 2015; Vilchèze et al., 2017). The inhibition of ATP synthase can sensitize resistant *Staphylococcus aureus* to polymyxin antibiotics (Vestergaard et al., 2017). Decreasing the efficiency of the pyruvate cycle and increasing fatty acid biosynthesis can increase resistance to ceftazidime in *Vibrio alginolyticus* (Liu et al., 2019). Mutations in the *tpiA* gene, a phosphotriester isomerase, reduced resistance to aminoglycoside antibiotics in *Pseudomonas aeruginosa* (Xia et al., 2020). The above findings suggest that microbial metabolism is closely related to antibiotic resistance.

However, the relationship between antibiotic resistance and microbial metabolism in the intestines of laying hens is unclear, which limits the prevention and control of antibiotic-resistant pollution and the healthy breeding of laying hens. The cecum is an important intestine that contains a large number of microbes that are closely related to the health of laying hens. In this study, the cecum contents of laying hens at different growth stages were first sampled. Then, the metagenome was used to analyze the bacterial community composition and ARG profile and to identify the dominant ARG hosts. The metabolome was used to analyze microbial metabolic profiles and relationships with ARGs and their hosts in the cecum. Finally, the effects of key metabolites on ARG and its host were verified. These results can provide a reference for the healthy breeding of laying hens and the prevention of antibiotic-resistant pollution.

## MATERIALS AND METHODS

### Slaughtering Experiment

**Figure 1:**
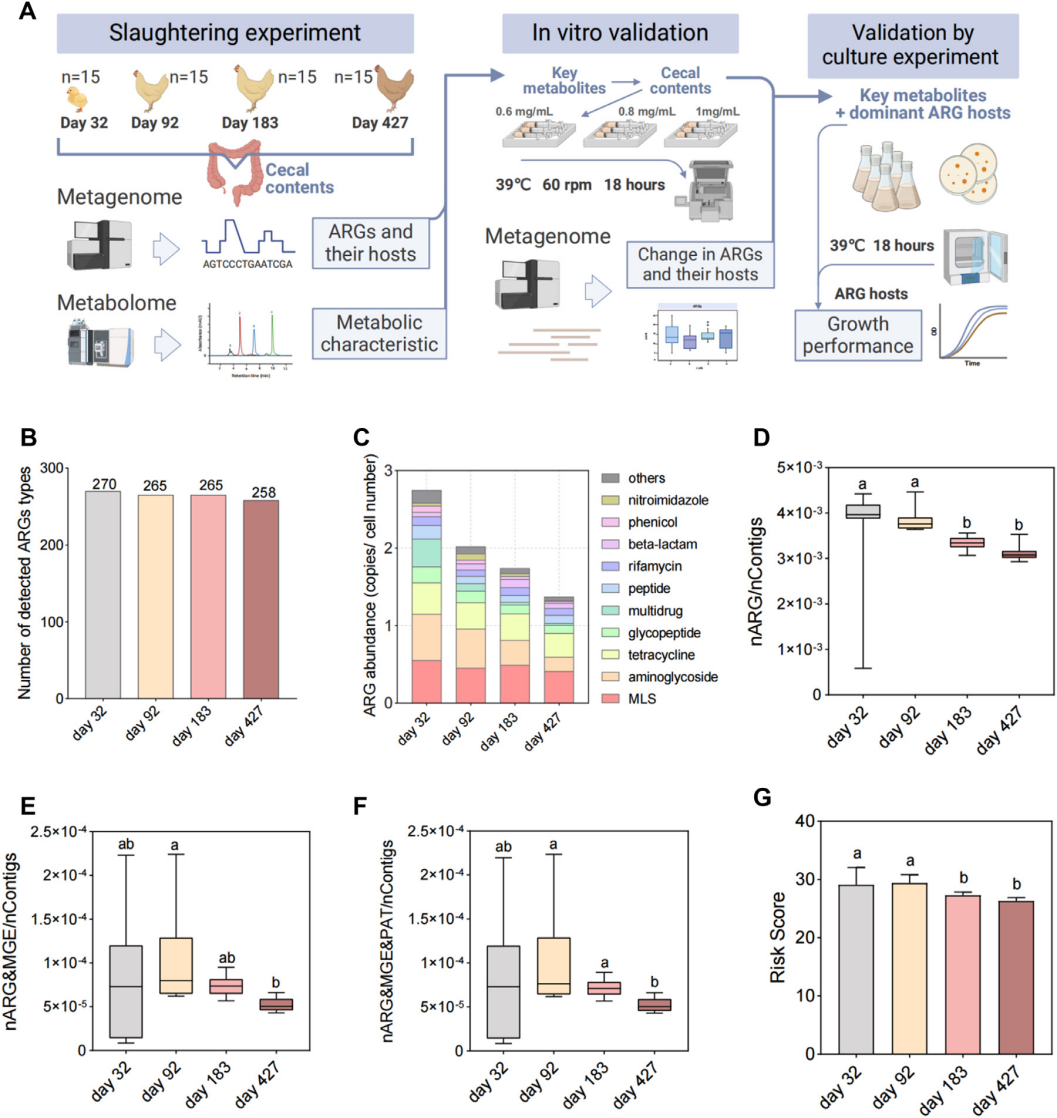
Antibiotic resistome in the intestine of laying hens. (A) The experimental flow of this study. The (B) number and (C) abundance of ARGs. (D, E and F) Proportion of contigs carrying ARGs, MGEs and PAT to total contigs. (G) Risk score of antibiotic resistome in the intestine of laying hens. Different letters indicate significant differences (*P* < 0.05).

Cecal contents were collected from 60 Hyland Grey laying hens, 15 each in the brooding stage (d 32), growing stage (d 92), laying stage (d 183), and late laying stage (d 427). After slaughtering the laying hens, the cecum contents were collected in freezing tubes, transferred to dry ice, and stored in a laboratory refrigerator at −80°C within 6 h. The ARGs were used to analyze the antibiotic resistome and microbial metabolism using the metagenome and metabolome (Figure 1A). This animal experiment was approved by the ethical center of South China Agricultural University.

### In Vitro Fermentation Experiment

The in vitro fermentation experiment was performed according to our previous study (Yang et al., 2021; Cai et al., 2023). First, laying hen feces were collected during the brooding period and kept in an anaerobic environment. Then, 100 g of feces was weighed, 300 mL of buffer solution was added, the filtrate was obtained by filtration through 4 layers of gauze, and the fermentation bacterial source was made by continuous passage of CO_2_ at 39°C. The experiment was designed with 4 groups, including 1 control group and 3 indole-3-lactic acid treatment groups, and 6 replicates were established for each group. Each group was supplemented with 200 mg of feed powder as fermentation substrate. The final concentrations of indole-3-lactic acid in the 3 treatment groups were 0.6, 0.8 and 1 mg/mL.

Next, 474 mL of ultrapure water, 237 mL of macronutrient solution, 237 mL of buffer solution, 0.21 mL of trace element solution, and 1.22 mL of bladed azurite solution were mixed in a 39°C water bath. Then, CO_2_ gas was passed for 10 min to remove dissolved oxygen from the solution. Then, 50 mL of reducing agent solution was added, and CO_2_ gas was continuously passed through the solution until the solution became colorless to obtain 1,000 mL of inoculum solution. Two hundred milliliters of fermentation bacterial source and 400 mL of inoculum solution were mixed as in vitro fermentation broth. Fermentation substrate and 10 mL of in vitro fermentation broth were added to the fermentation tubes, which were sealed and placed on a shaker after the air was exhausted. The samples were incubated at 39°C and 60 rpm for 18 h. The fermentation tubes were then placed on ice to terminate fermentation and stored in a −80°C freezer for metagenomic analysis.

### Single-Bacteria Culture Experiment

The *Escherichia coli* used in the experiment were isolated and identified in the intestine of laying hens in the pre-experiment. LB broth media with indole-3-lactic acid at concentrations of 0, 0.1, 0.2, 0.3, 0.4, 0.5, 0.6, 0.7, 0.8, 0.9, and 1 mg/mL were prepared. The plates were added to 96-well polystyrene plates in order of concentration. *Escherichia coli* was diluted 10^−6^ and then inoculated at a 1% concentration. The plates were then placed in an incubator at 39°C for 18 h. The OD600 was detected by an enzyme marker. The growth curve (600 OD) of *Escherichia coli* in broth medium supplemented with 0.8 mg/mL indole-3-lactic acid was determined for 48 h. Additionally, 2 μL of *Escherichia coli* was inoculated on agar medium supplemented with 0.8 mg/mL indole-3-lactic acid and incubated for 24 h, after which the colony area was detected.

### Metagenomic Sequencing and Analysis

A total of 84 samples were used for metagenomic sequencing, including 60 cecal contents and 24 fermentation broths. Sample DNA was extracted using the QIAamp PowerFecal Pro DNA Kit (Qiagen, Germany). DNA concentration, purity and integrity were evaluated using a Qubit Utra-Micro spectrophotometer (Qubit 3, Thermo Fisher Scientific) and gel imaging (2500, Tanon, China). After quality control, DNA samples were used to construct DNA libraries at Novogene (Beijing, China) using the NEBNext Ultra DNA Library Prep Kit for Illumina (NEB). Then, sequencing was performed on an Illumina NovaSeq X Plus platform. The quality of the raw sequence was controlled using Trimmomatic v_0.33 (SLIDINGWINDOW:4:20, MINLEN:50) (Bolger et al., 2014), and chicken DNA was removed from the chicken genome (GCF_016699485.2) using Bowtie 2 (v_0.1) software (Langmead and Salzberg, 2012). The clean sequencing data were assembled using MEGAHIT (v1.2.9) software with the default parameters (kmer-lists 21, 29, 39, 59, 79, 99, 119, and 141) (Li et al., 2015). Next, Prodigal v2.63 was used to predict genes (Hyatt et al., 2010). CD-HIT (v4.8.1) and Salmon software were used to cluster genes and calculate gene abundance, respectively, with default parameters (Li and Godzik, 2006, Patro et al., 2017).

The bacterial composition was analyzed with Kraken 2 software (Wood and Salzberg, 2014). DIAMOND (v2.0.15.153) was used to identify antibiotic resistance genes (**ARGs**) based on the Comprehensive Antibiotic Resistance Database (CARD v3.2.5) with the following parameters: -e 1e-6, –query-cover 70, and –id 60. ARGs-OAP v3.2 was used to calculate the abundance of ARGs (normalized to copies per prokaryotic cell number) (Buchfink et al., 2015, Yang et al., 2023). The MetaCompare pipeline was used to identify the number of contigs carrying ARGs, mobile genetic elements (**MGEs**) and pathogen (**PAT**) sequences (Oh et al., 2018). According to the number and proportion of these contigs, the resistome risk of each sample was calculated using the MetaCompare pipeline (https://github.com/minoh0201/MetaCompare).

### Metagenomic Binning and Identification of ARG Hosts

Contigs longer than 2000 kb were selected for metagenomic binning. Metagenome-assembled genomes (**MAGs**) were generated using MetaBAT2, MaxBin and CONCOCT in the MetaWRAP pipeline (Uritskiy et al., 2018). CheckM software was used to assess the completeness and contamination of these MAGs (Parks et al., 2015). The MAGs with a completeness greater than 50% and contamination less than 10% were used for subsequent analysis. The taxonomy of these MAGs was classified based on the Genome Taxonomy Database (**GTDB**, R214) using GTDB-Tk (v 2.3.2) (Parks et al., 2022). ARGs in these MAGs were identified with DIAMOND software.

### Metabolomics Analysis

Sixty samples of cecal contents were subjected to untargeted metabolomics. The samples were placed in EP tubes, and 300 μL of 80% methanol in water was added. The samples were snap frozen in liquid nitrogen for 5 min, thawed on ice, vortexed for 30 s, sonicated for 6 min, and centrifuged at 5000 rpm at 4°C for 1 min, after which the supernatant was transferred to a new centrifuge tube and lyophilized into a dry powder. The sample was dissolved in 10% methanol by volume. The samples were detected by liquid chromatography‒mass spectrometry (LC‒MS) at Novogene (Beijing, China).

Blank group and QC group (equal volume mixed samples) was set up to balance the instrument status and remove background ions. The liquid chromatography platform used was a Thermo Vanquish (Thermo Fisher Scientific). An ACQUITYUPLC®HSST3 (2.1×150 mm, 1.8 µm) (Waters, Milford, MA) column was used with a flow rate of 0.25 mL/min, a column temperature of 40°C, and an injection volume of 2 μL. In positive ionization mode, the mobile phases were 0.1% formic acid in acetonitrile (C) and 0.1% formic acid in water (D). The gradient elution program was as follows: 0∼1 min, 2% C; 1∼9 min, 2%∼50% C; 9∼12 min, 50%∼98% C; 12∼13.5 min, 98% C; 13.5∼14 min, 98%∼2% C; and 14∼20 min, 2% C. In negative ionization mode, the mobile phases were acetonitrile (A) and 5 mM ammonium formate water (B). The gradient elution program was as follows: 0∼1 min, 2% A; 1∼9 min, 2% to 50% A; 9∼12 min, 50% to 98% A; 12∼13.5 min, 98% A; 13.5∼14 min, 98% to 2% A; and 14∼17 min, 2% A. A ThermoQ Exactive mass spectrometry detector (Thermo Fisher Scientific) was used. The positive ion spray voltage was 3.50 kV, the negative ion spray voltage was -2.50 kV, the sheath gas was 30 arb, and the auxiliary gas was 10 arb. The capillary temperature was 325°C, and a primary full scan was performed at a resolution of 70,000, with a primary ion scan range of 81∼1000 m/z. HCD was used for secondary cleavage, with a collision voltage of 30% and a secondary resolution of 17,500. The first 10 ions of the acquired signal were fragmented, while dynamic exclusion was used to remove unnecessary MS/MS information. The downloaded data were organized and compared with the mzCloud (https://www.mzcloud.org/), mzVault and Masslist databases. Background ions were removed from the blank samples, and the raw quantitative results were normalized to finally obtain the relative abundance of metabolites.

### Data Analysis and Presentation

The data were prepared in the WPS office (v12.1). SPASS (v22.0) was used for significance and correlation analyses. R software (v4.2.2) was used for plotting the PCA results. A phylogenetic tree of the MAGs was constructed with Interactive Tree Of Life (iTOL, v6.74) (Letunic and Bork, 2011). GraphPad Prism 8 software was used for the other plots. Adobe Illustrator 22.1 was used for the graphic layout.

## RESULTS

### Antibiotic Resistome in the Laying Hen Intestine

The greatest number of ARGs were detected in the cecum of laying hens on d 32 (270 ARGs), followed by 265 ARGs on d 92 and 183, and the lowest number of ARGs were detected on d 427, with 258 ARGs (Figure 1B). The total ARG abundance was similar to the ARG number (Figure 1C). The highest total abundance of ARGs, 2.74 ± 1.37 copies/cell, was detected in the cecum contents of laying hens on d 32. The total abundances of ARGs on d 92 and 183 were 2.02 ± 0.17 and 1.74 ± 0.13 copies/cell number, respectively. The lowest total abundance of ARGs, 1.37 ± 0.07 copies/cell number, was detected on d 427. This indicated a decreasing trend in the total abundance of ARGs during the growth of laying hens. Among these ARGs, the highest abundance of ARGs was detected for the MLS resistance genes, followed by the aminoglycoside, tetracycline, glycopeptide, and multidrug resistance genes. This also suggested that the abundance of ARGs in the intestine changed with different growth periods, but the abundance of the main components of the ARGs did not change significantly. We then analyzed the contigs carrying ARGs, MGEs and PAT to calculate the ARG risk score. The ratio of contigs carrying ARGs, the ratio of contigs carrying both ARGs and MGEs, and the ratio of contigs carrying both ARGs, MGEs and PAHs tended to decrease gradually with growth stage (Figures 1D, 1E and 1F), which was similar to the results of the number and abundance of ARGs detected. Finally, the ratio of the above contigs was analyzed for the antibiotic resistome risk score using the MetaCompare pipeline, and it was found that the risk scores of the resistome also gradually decreased (Figures 1G). This may be due to the use of more antibiotic drugs in the pregrowth period and the ban on antibiotics in the later laying period.

### Hosts of Antibiotic Resistance Genes in the Laying Hen Intestine

**Figure 2:**
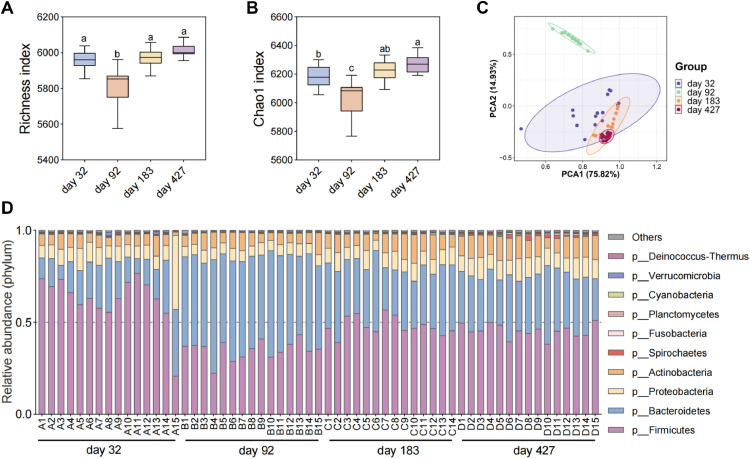
Bacterial community composition in the intestine of laying hens. (A) Richness index and (B) Chao1 index of bacterial communities. (C) Principal component analysis of bacterial community composition. (D) Relative abundance of bacterial communities (phylum). Different letters indicate significant differences (*P* < 0.05).

**Figure 3:**
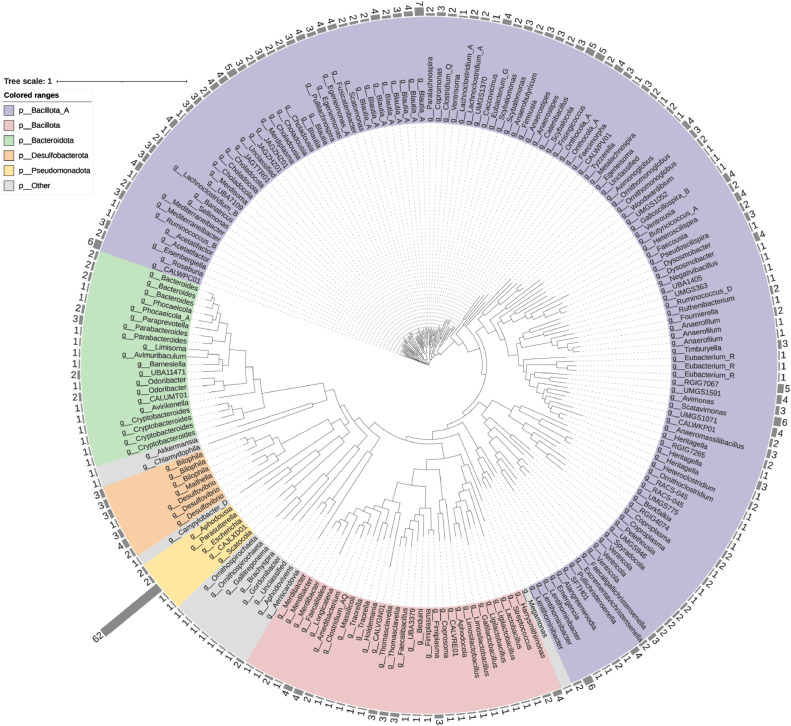
Phylogenetic tree of the ARG host. The genomes of these ARG hosts were obtained by metagenome binning (>90% completeness and <5% contamination). The numbers in the figure indicate the number of ARGs carried.

To identify ARG hosts, we analyzed the microbial diversity and composition in the intestines of laying hens. The alpha diversity index, Richness index and Chao1 index were the highest on d 472 and were significantly lower on d 92 than those in the other stages (Figures 2A and 2B). The PCA results also revealed that the microbial composition on d 92 was significantly different from the microbial composition in other periods (Figure 2C). The highest relative abundance of Bacteroidetes was found in the cecum of laying hens on d 92, followed by Firmicutes, Proteobacteria and Actinobacteria (Figure 2D). The highest abundance of Firmicutes, followed by Bacteroidetes, Proteobacteria and Actinobacteria, was observed in the other stages. The above results indicated that the microbial composition at d 92 differed significantly from that at other stages, but overall, Firmicutes and Bacteroidetes were the dominant phyla at all stages. At the genus level, *Alistipes* and *Bacteroides* had the highest relative abundances, followed by *Lactobacillus, Faecalibacterium* and *Lachnoclostridium* (Supplementary Figure S1). The abundances of genera such as *Escherichia* were found to be significantly positively correlated with the total number of ARGs (*P* < 0.01) (Supplementary Table S1). Then, the ARG hosts were mined via metagenome binning. In total, 1142 MAGs were obtained from the intestines of laying hens with GC contents of 0.236 to 0.712, completeness >50% and contamination <10% (Supplementary Table S2). Among them, 724 MAGs carried ARGs and were considered ARG hosts. To obtain more accurate information about ARG hosts, we analyzed these MAGs and screened them to obtain 200 ARG hosts with greater than 90% completeness and less than 5% contamination (Figure 3). Bacillota_A (Firmicutes_A) and Bacillota (Firmicutes) were the most abundant phyla, followed by Bacteroidetes. This result was similar to that of the bacterial composition (Figure 2D). In addition, *Escherichia coli,* which carried 62 ARGs, was found to be the dominant ARG host. All other hosts carried fewer than 10 ARGs.

### Relationship Between Microbial Metabolism and Antibiotic Resistome

**Figure 4:**
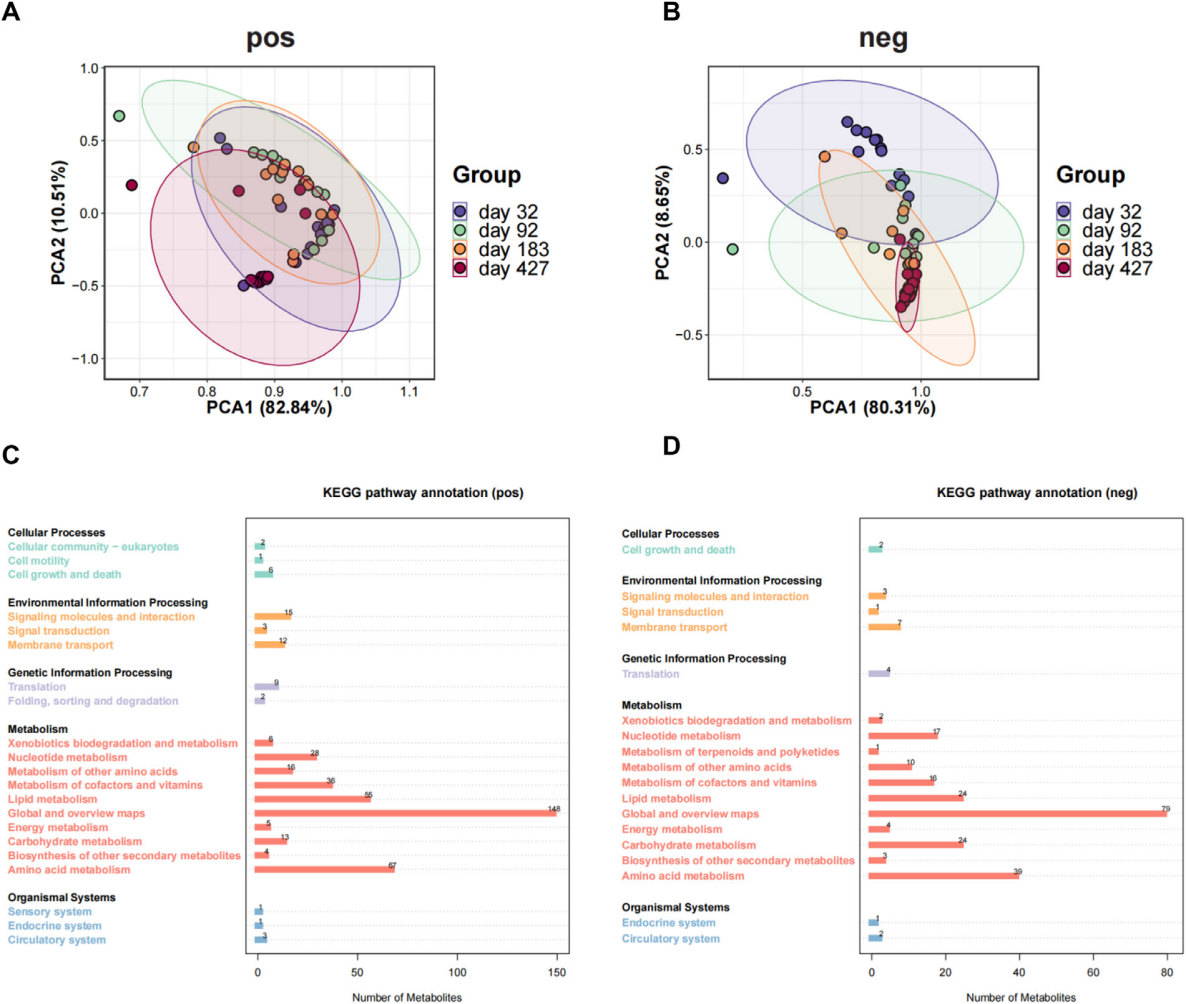
Microbial metabolism in the intestine of laying hens. Principal component analysis of microbial metabolites in (A) positive and (B) negative ion modes. KEGG pathway annotation of microbial metabolites in (C) positive and (D) negative ion modes.

The microbial metabolism of the cecal contents of laying hens was analyzed. In positive ion mode, the differences in intestinal microbial metabolism at different periods were not significant (Figure 4A). In negative ion mode, the difference in metabolite composition was significant on d 32 and 427, while the difference in metabolite composition was not significant in the other periods (Figure 4B). There were 433 and 239 microbial metabolic pathways annotated in the positive and negative ion modes, respectively (Figure 4, Figure 4D). The pathway enriched in metabolism was the most abundant, followed by environmental information processing and genetic information processing. The different types of metabolites were correlated with the different types of ARGs in the top 10 most abundant genes (Supplementary Figure S2). Organic oxygen compounds and organic nitrogen compounds were significantly positively correlated with all the ARGs (*P* < 0.05). Other metabolites were significantly positively or negatively correlated with different types of ARGs. We also found that indole and its derivatives were significantly negatively correlated with aminoglycoside, tetracycline, glycopeptide and phenicol resistance genes (*P* < 0.05) (Table 1). The abundance of indole-3-lactic acid was linearly correlated with the abundance of the tet(W), tet(O/W), tet(W/N/W), rpoB2, lnuC, and *LlmA_23S_CLI* resistance genes (Supplementary Figure S3). The above results suggest a close association between microbial metabolism and the antibiotic resistome in intestine of laying hens, with indole-3-lactic acid as the key metabolite.

**Table 1:** Correlation of different types of ARGs with indole and its derivatives (Spearman).

ARGs	R	Sig.
MLS	−0.05	*P* = 0.70
Aminoglycoside	−0.35	*P =* 0.01
Tetracycline	−0.35	*P* = 0.01
Glycopeptide	−0.34	*P* = 0.01
Multidrug	−0.21	*P* = 0.11
Peptide	−0.28	*P* = 0.03
Rifamycin	−0.01	*P* = 0.94
Beta-lactam	0.06	*P* = 0.65
Phenicol	−0.38	*P* = 0.00
Nitroimidazole	−0.11	*P* = 0.40

### Validation of the Effects of Key Metabolites on the Antibiotic Resistome

**Figure 5:**
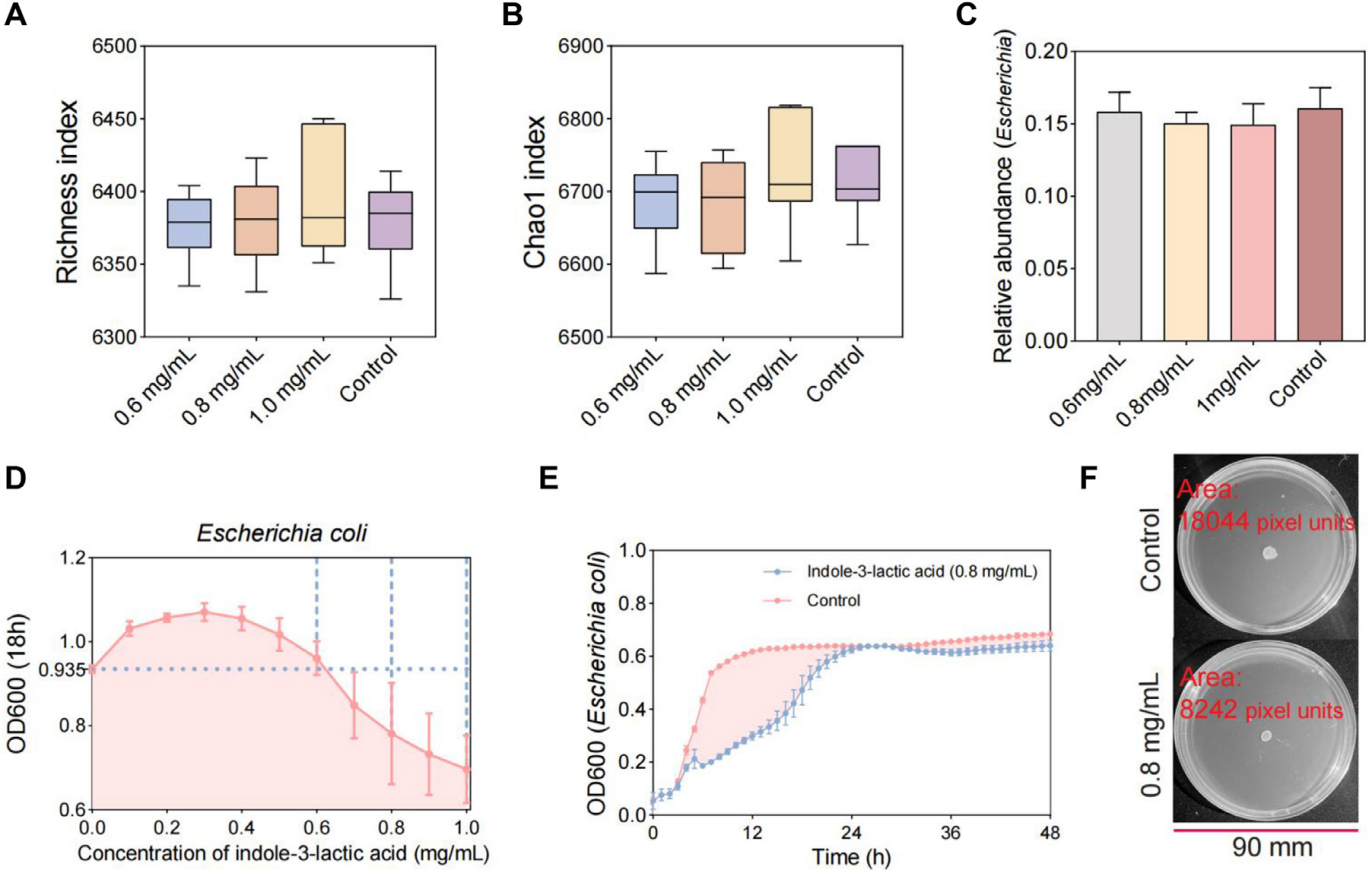
Effect of key metabolite on bacterial community composition. Effect of the key metabolite (indole-3-lactate) on the (A) richness index and (B) chao1 index of bacterial communities in in vitro fermentation experiment. (C) Effect of indole-3-lactic acid on the dominant ARG host (*Escherichia*) in in vitro fermentation experiment. (D and E) Effect of indole-3-lactate on the growth of *Escherichia coli* in culture experiment. (F) Effect of indole-3-lactate on the colony area of *Escherichia coli*.

**Figure 6:**
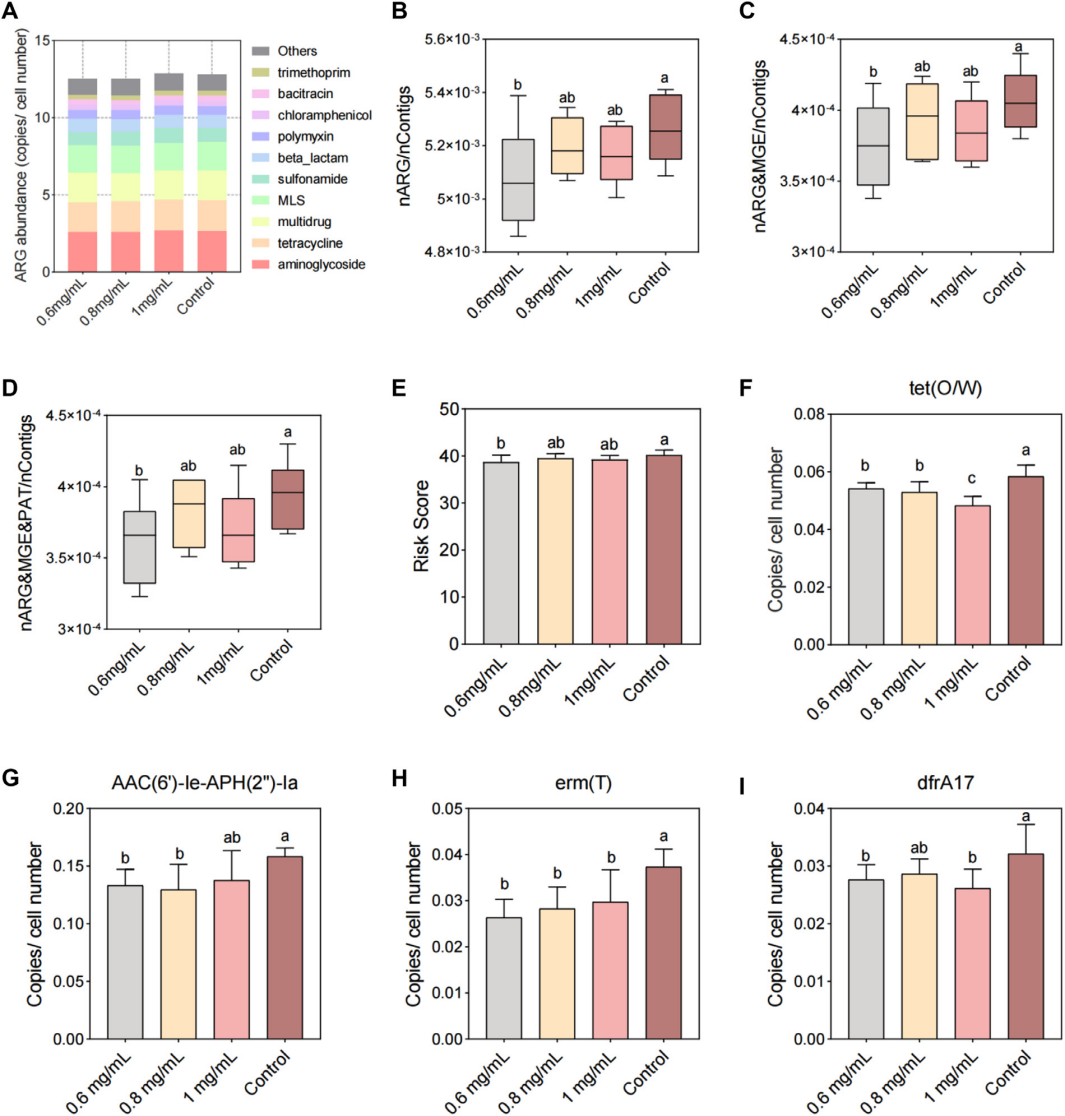
Effect of key metabolite on antibiotic resistome. (A) Effect of indole-3-lactate on ARG abundance in in vitro fermentation experiment. (B, C and D) Proportion of contigs carrying ARGs, MGEs and PAT to total contigs. (E) Risk score of antibiotic resistome in in vitro fermentation experiment. (F, G, H and I) The abundance of *tet(O/W), ACC(6′)-Ie-APH(2′’)-Ia, erm(T)* and *afrA17* resistance genes. Different letters indicate significant differences (*P* < 0.05).

We further verified the effect of the key metabolite indole-3-lactic acid on the antibiotic resistome. In this study, the feces of brooding laying hens with the greatest risk to the resistome and greatest amount of indole-3-lactic acid were selected for in vitro fermentation experiment. The addition of 0.6, 0.8 and 1 mg/mL indole-3-lactic acid had no significant effect on the microbial Richness index and Chao1 index in the fermentation broth (Figures 5A and 5B). This indicated that indole-3-lactic acid had no significant effect on the Alpha diversity of the microbial community. The addition of indole-3-lactic acid had no significant effect on the microbial composition in the fermentation broth (Supplementary Figure S4). However, the abundance of *Escherichia coli* was lower in the group supplemented with indole-3-lactic acid than in the control group (Figure 5C). The lowest *Escherichia coli* abundance was detected in the 0.8 mg/mL and 1 mg/mL groups. This indicates that although the key metabolite indole-3-lactic acid had no significant effect on microbial diversity or composition in the fermentation broth, it was able to reduce the abundance of the dominant ARG host (*Escherichia coli*). Then, we conducted single-bacteria culture experiments and found that the growth density of *Escherichia coli* tended to increase and then decrease as the concentration of indole-3-lactic acid added increased (Figure 5D). The concentration of *Escherichia coli* significantly decreased at indole-3-lactic acid concentrations of 0.8 mg/mL and 1 mg/mL. We used 0.8 mg/mL indole-3-lactic acid to culture *Escherichia coli* and found that its growth rate and density were inhibited (Figure 5E). The colony area of *Escherichia coli* in the 0.8 mg/mL indole-3-lactic acid treatment group was significantly smaller than that in the control group (Figure 5F). These results suggested that the key metabolite indole-3-lactic acid inhibited the growth of the dominant ARG host (*Escherichia coli*). We then analyzed the composition of ARGs in the fermentation broth. The abundance of ARGs in the 0.6 mg/mL and 0.8 mg/mL indole-3-lactic acid groups was lower than that in the control group (Figure 6A). The results also revealed that the ratio of contigs carrying ARGs, the ratio of contigs carrying both ARGs and MGEs, and the ratio of contigs carrying both ARGs, MGEs, and PAHs were greater in the indole-3-lactic acid-treated group than in the control group (Figures 6B, 6C, and 6D). The risk score of the antibiotic resistome in the indole 3 lactic acid-treated group was also lower than that in the control group (Figure 6E). In addition, we found that the abundances of the tet(O/W), ACC(6′)-Ie-APH(2′')-Ia, erm(T), and dfrA17 resistance genes were significantly lower in the indole-3-lactic acid-treated group than in the control group (Figures 6F, 6G, 6H and 6I). These results suggested that key metabolites can inhibit the growth of dominant ARG hosts, which in turn reduces ARG abundance and risk.

## DISCUSSION

In this study, we found that the total abundance of ARGs in the cecal contents of laying hens was the highest during the brooding stage, the average total abundance of ARGs in the cecal contents of laying hens tended to decrease with the growth of laying hens, and the total abundance of ARGs in the cecal contents of laying hens was the lowest in the late laying stage. This may be because laying hens are weak and susceptible to disease during the brooding period, requiring more antibiotics, as well as because antibiotics are not allowed for laying hens during the laying period. A similar study revealed that the relative abundance of ARGs in manure samples from laying hens at different feeding stages decreased in the following order: brooding period, late laying period, growing period, early laying period, and peak laying period (Zhu et al., 2021). The risk score for antibiotic resistance in the cecum contents was greater in the brooding and growing stages than in the laying and late laying stages. The main ARGs in the cecum of laying hens were MLS, aminoglycoside, tetracycline, glycopeptide, and multidrug resistance genes. Previous studies have also identified tetracycline, aminoglycoside and macrolide resistance genes as the most common ARG types in the chicken gut (Hastings et al., 2004). However, there were large differences in abundance between the major ARG subtypes in laying hens at various stages, mainly ANT(6)-Ib, LimA_23S_CLI, APH(3′)-IIIa, ugd, tet(Q), rpoB2, ErmF and tet(O/W). ARGs in the intestines of laying hens may be related to nonantibiotic factors, such as surrounding environmental bacteria, in addition to medication (Bengtsson-Palme et al., 2018, Zhu et al., 2023). In addition, the present study revealed that *Escherichia* are potential host bacteria for major ARGs in the intestinal tract of laying hens. *Escherichia* are common opportunistic pathogenic genera in the gastrointestinal tract of chickens. Some studies have isolated *Escherichia coli* from broiler chickens that are resistant to at least 4 antibiotics (Ribeiro et al., 2023). It has also been shown that *Escherichia coli* strains isolated from chicken harbor ARGs such as tet(A), tet(B), dfrA1, qnrA, catA1, cmlA, sul1 and ere(A).

The normal metabolism of gut microbes plays an important role in ensuring host health. Recent studies have shown that microbial metabolic adaptation may involve a class of independent antibiotic resistance mechanisms and that mutations in core metabolic genes can lead to the development of antibiotic resistance (Lopatkin et al., 2021). This suggests that bacterial metabolic genes may be involved in the regulation of bacterial antibiotic resistance and that changes in metabolic pathways can affect bacterial antibiotic resistance. In this study, *Escherichia coli* was found to be the dominant ARG host in the intestines of laying hens. The metabolite indole-3-lactic acid reduced microbial community diversity and inhibited the growth of the ARG host *Escherichia coli*. In addition, indole-3-lactic acid was effective in reducing the abundance of the AAC(6′)-Ie-APH(2′')-Ia, tet(O/W), erm(T) and dfrA17 resistance genes. It has also been found that indole production reduces the resistance of enzyme-producing *Lysostaphylium* to various antibiotics, such as ampicillin and kanamycin (Han et al., 2011). Thus, microbial metabolism in the intestines of laying hens is associated with changes in antibiotic resistance, and the key metabolite is indole-3-lactic acid. In addition, indole-3-lactic acid has a positive effect on the animal gut in addition to its possible association with bacterial antibiotic resistance (Sakurai et al., 2019).

In recent years, the contamination of antibiotic-resistant bacteria and ARGs during farming has become a growing concern. The prevention and control of drug-resistant pollution during farming has become urgent. This study explored the association between bacterial metabolism and antibiotic resistance to provide a reference for the prevention and control of antibiotic-resistant pollution. Our findings suggest that microbial metabolism is closely related to antibiotic resistance and represents a breakthrough in antibiotic resistance prevention. In this study, indole-3-lactic acid was found to inhibit the growth of the ARG host *Escherichia coli* and has the potential to reduce resistome risk in the intestines of laying hens. It has been shown that by modulating metabolism, antibiotic-resistant bacteria can be reconverted to antibiotic-sensitive susceptible bacteria (Martínez and Rojo, 2011). It has also been shown that carbon sources and cellular respiration influence bacterial susceptibility to antibiotics (Vitko et al., 2015; Vilchèze et al., 2017). Inhibition of a central metabolic enzyme, ATP synthase, can sensitize antibiotic-resistant *Staphylococcus aureus* to polymyxin antibiotics (Vestergaard et al., 2017). Decreasing the efficiency of the pyruvate cycle and increasing the synthesis of fatty acids can increase resistance to ceftazidime in *Vibrio alginolyticus* (Liu et al., 2019). Mutations in the tpiA gene, a phosphotriester isomerase, reduce resistance to aminoglycoside antibiotics in *Pseudomonas aeruginosa* (Xia et al., 2020).

In summary, it is possible to prevent and control antibiotic-resistant pollution from the perspective of microbial metabolism. For example, reagents and drugs that regulate the metabolism of environmental microorganisms can be developed to treat antibiotic-resistant pollution. However, more problems remain to be solved to achieve this objective. For example, environmental microorganisms are complex systems, and the administration of regulatory reagents and drugs not only inhibits resistant microorganisms but also may affect other microorganisms, even beneficial ones. Whether the widespread use of these metabolites will create new contamination problems also needs to be further evaluated. In addition, the current application cost of this method is high, its prospects for application in various environmental conditions, such as culture, are weak, and it may have greater application value in various environments, such as clinical settings. In any case, with the development of technology, these problems will likely be gradually solved.

## CONCLUSION

A slaughtering experiment, an in vitro fermentation experiment and a single-bacteria culture experiment were carried out in this study. There were different types of ARGs in the intestines of laying hens, and the risk scores of ARGs tended to decrease with growth stage. In addition, *Escherichia coli*, which carried 62 ARGs, was found to be the dominant ARG host. Metabolomics revealed that indole and its derivatives, such as indole-3-lactic acid, were negatively correlated with a variety of ARGs. Moreover, indole-3-lactic acid reduced the abundance and risk of multiple ARGs in the intestine and inhibited the growth of the ARG host *Escherichia coli*. In the context of high concern about intestinal microbial metabolism and antibiotic resistance, this is the first study to focus on the relationship between intestinal microbial metabolism and antibiotic resistance in laying hens. Our findings have important implications for healthy farming and antibiotic resistance control.

## CRediT authorship contribution statement

**Yilin Yuan:** Formal analysis, Investigation. **Chunhao Mo:** Investigation. **Feng Huang:** Investigation, Methodology. **Xindi Liao:** Investigation, Writing – review & editing, Funding acquisition. **Yiwen Yang:** Conceptualization, Investigation, Methodology, Writing – original draft, Project administration, Funding acquisition.

## DISCLOSURES

The authors declare that they have no known competing financial interests or personal relationships that could have appeared to influence the work reported in this paper.

## Appendix Supplementary materials

Image, application 1

## Footnotes

Supplementary material associated with this article can be found in the online version at doi:10.1016/j.psj.2024.104138.
