# Supplementary material for: Microbial metabolism affects the antibiotic resistome in the intestine of laying hens
Source: Poult Sci. 2024 Jul 29;103(10):104138. doi: 10.1016/j.psj.2024.104138 (PMC11375135; doi:10.1016/j.psj.2024.104138)
Supplement: Supplementary file 1 [file mmc1.docx]

**Microbial metabolism affects the antibiotic resistome in the intestine of laying hens**

Yilin Yuan^1^, Chunhao Mo^1^, Feng Huang^2^, Xindi Liao^1^, Yiwen Yang^1^*,

^1^ Guangdong Provincial Key Laboratory of Agro-Animal Genomics and Molecular Breeding, State Key Laboratory of Swine and Poultry Breeding Industry, College of Animal Science, South China Agricultural University, Guangzhou, China

^2^ School of Biological Engineering, Henan University of Technology, Zhengzhou, China

***Corresponding author:**

Yiwen Yang, Ph.D.

South China Agricultural University

E-mail: [yiweny@foxmail.com](mailto:yiweny@foxmail.com)/[yiweny@scau.edu.cn](mailto:yiweny@scau.edu.cn)


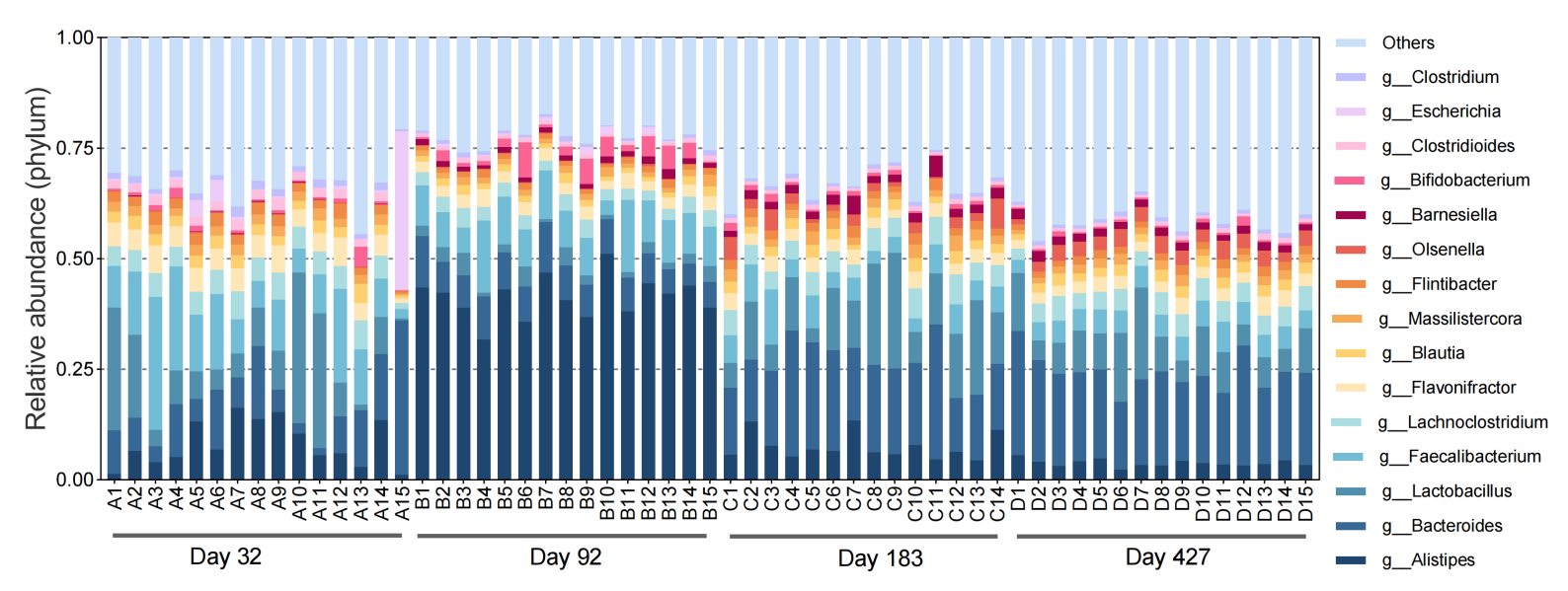


Fig S1. Relative abundance of bacterial communities (genus) in the intestines of laying hens


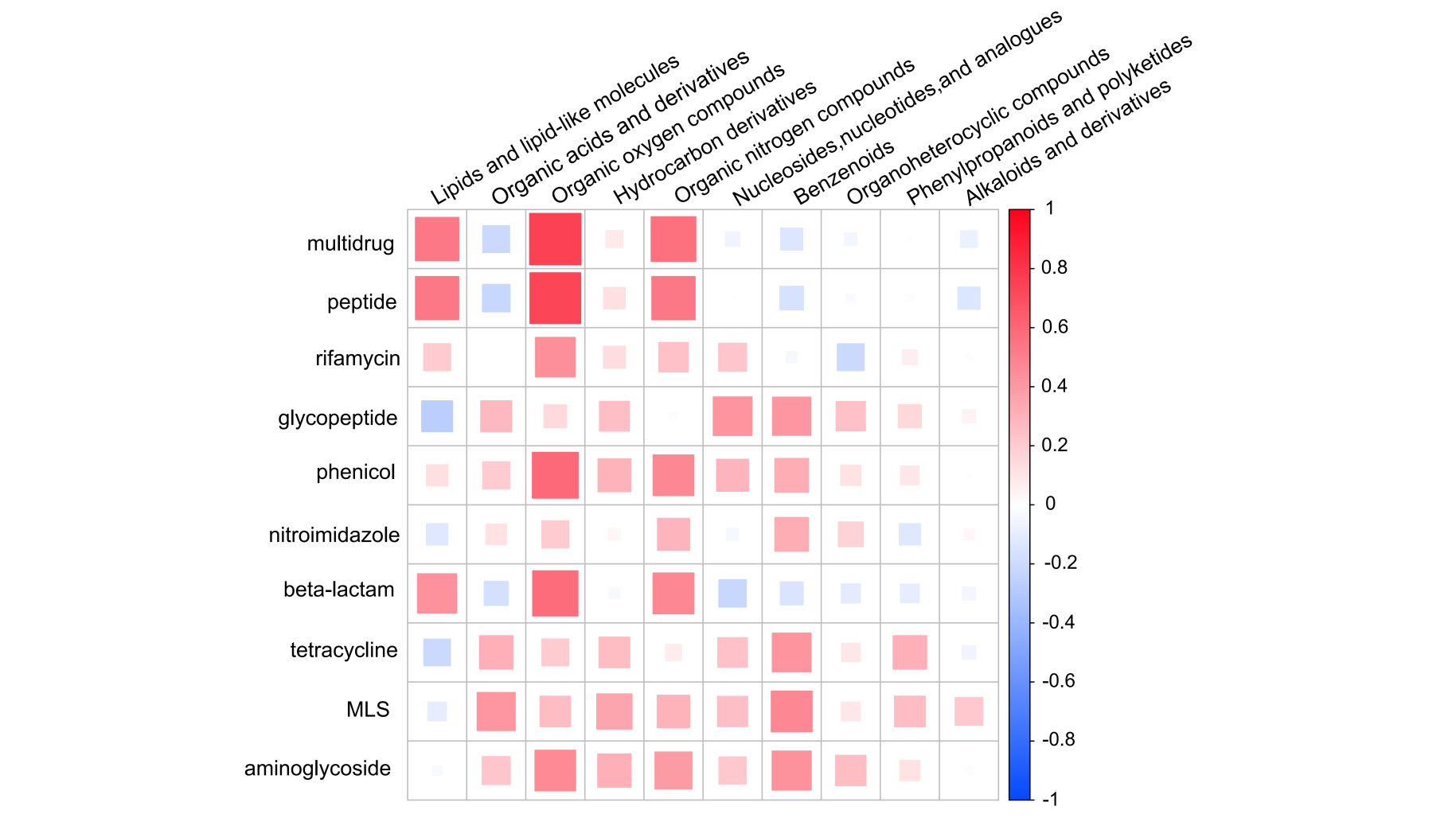


Fig S2. Correlation between metabolites and ARGs (Spearman)


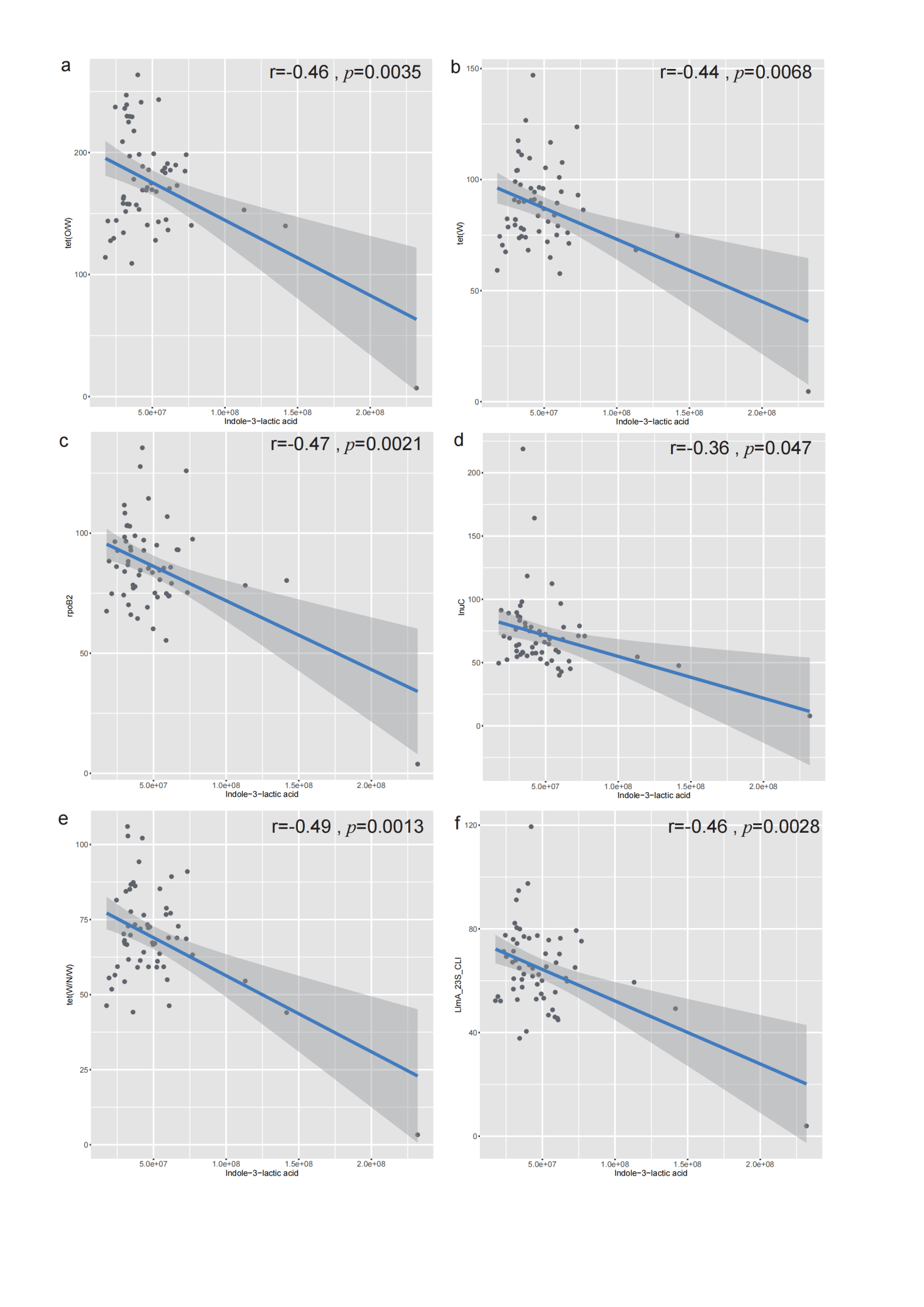


Fig S3. Linear relationship between indole-3-lactic acid and ARGs


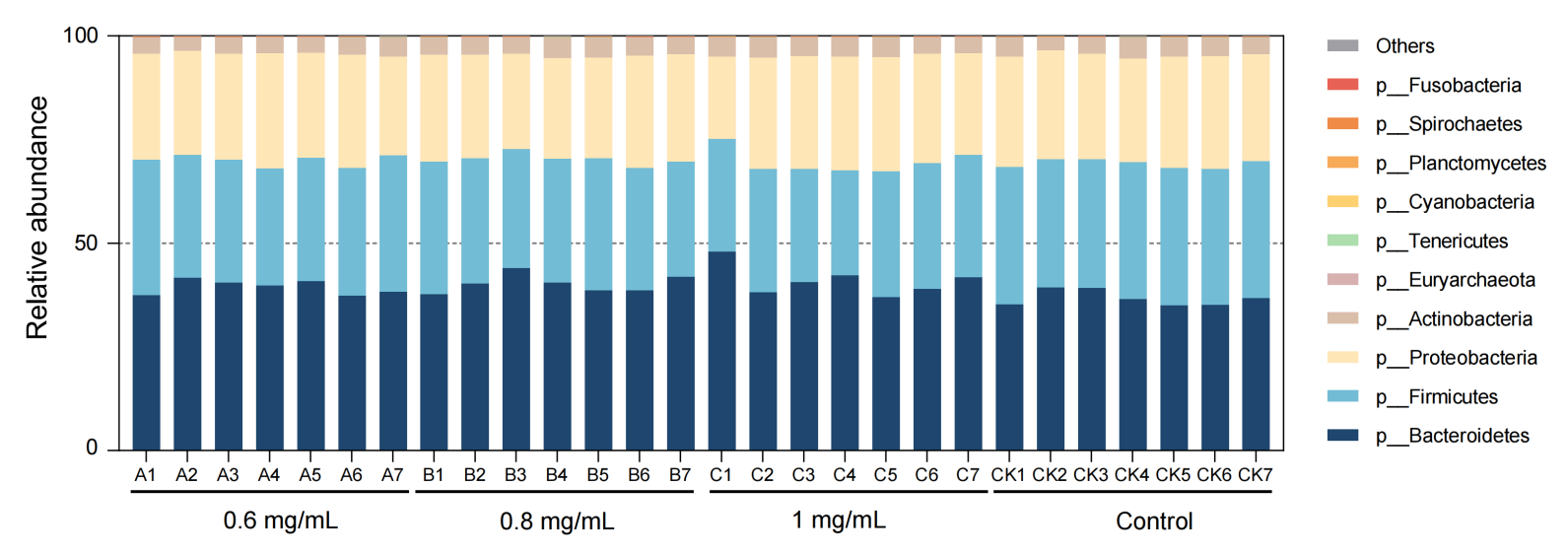


Fig S4. Relative abundance of bacterial communities (phylum) in fermentation broth

Table S1 Correlation between genera and total ARG abundance (Spearman)

| Genera | R | Sig. |
| --- | --- | --- |
| *Escherichia* | 0.91 | *P＜0.01* |
| *Enterococcus* | 0.85 | *P＜0.01* |
| *Enterocloster* | 0.25 | *P＞0.05* |
| *Clostridioides* | 0.17 | *P＞0.05* |
| *Faecalibacterium* | 0.14 | *P＞0.05* |
| *Eubacterium* | 0.13 | *P＞0.05* |
| *Clostridium* | 0.06 | *P＞0.05* |
| *Flavonifractor* | 0.04 | *P＞0.05* |
| *Streptococcus* | 0.04 | *P＞0.05* |
| *Alistipes* | 0.04 | *P＞0.05* |
| *Ruthenibacterium* | 0.01 | *P＞0.05* |
| *Roseburia* | 0.01 | *P＞0.05* |
| *Bacteroides* | 0.01 | *P＞0.05* |

Table S2 MAG stats

| MAGs | completeness | contamination | GC | N50 | size |
| --- | --- | --- | --- | --- | --- |
| bin.1 | 91.91 | 0.557 | 0.437 | 39246 | 4127141 |
| bin.10 | 76.62 | 5.592 | 0.646 | 8319 | 1746954 |
| bin.100 | 81.08 | 0.37 | 0.436 | 6936 | 970951 |
| bin.1000 | 73.15 | 0.692 | 0.629 | 16356 | 1238894 |
| bin.1001 | 89.5 | 5.241 | 0.666 | 7451 | 1786037 |
| bin.1002 | 94.25 | 1.775 | 0.641 | 12407 | 2854188 |
| bin.1003 | 67.62 | 3.914 | 0.267 | 15941 | 1422078 |
| bin.1004 | 86.31 | 1.005 | 0.573 | 53745 | 1708611 |
| bin.1005 | 50.79 | 4.127 | 0.262 | 6795 | 919528 |
| bin.1006 | 84.93 | 9.247 | 0.542 | 5196 | 1493623 |
| bin.1007 | 66.54 | 1.006 | 0.624 | 9042 | 1689708 |
| bin.1008 | 64.1 | 3.448 | 0.475 | 10315 | 1460923 |
| bin.1009 | 71.19 | 1.083 | 0.636 | 10400 | 1451411 |
| bin.101 | 82.55 | 3.83 | 0.645 | 4971 | 1801826 |
| bin.1010 | 84 | 2.237 | 0.497 | 25941 | 1615756 |
| bin.1011 | 67.75 | 6.845 | 0.648 | 14213 | 1964988 |
| bin.1012 | 52.63 | 5.263 | 0.286 | 5633 | 541174 |
| bin.1013 | 87.68 | 0 | 0.28 | 14148 | 1095988 |
| bin.1014 | 63.26 | 1.875 | 0.56 | 5231 | 1343664 |
| bin.1015 | 85.83 | 4.461 | 0.613 | 6743 | 1498680 |
| bin.1016 | 92.28 | 0.846 | 0.441 | 15729 | 2912173 |
| bin.1017 | 95.7 | 2.973 | 0.479 | 14196 | 3186873 |
| bin.1018 | 50.87 | 7.894 | 0.27 | 4010 | 937221 |
| bin.1019 | 71.32 | 6.741 | 0.25 | 6537 | 1066091 |
| bin.102 | 100 | 0 | 0.482 | 170903 | 1995062 |
| bin.1020 | 79.17 | 0.549 | 0.38 | 13900 | 735343 |
| bin.1021 | 94.75 | 2.908 | 0.409 | 17856 | 1585352 |
| bin.1022 | 80.96 | 1.575 | 0.277 | 6319 | 1167685 |
| bin.1023 | 79.98 | 6.518 | 0.583 | 8273 | 1743292 |
| bin.1024 | 93.73 | 0 | 0.51 | 43639 | 1774247 |
| bin.1025 | 72.6 | 3.169 | 0.567 | 4587 | 1341367 |
| bin.1026 | 63.21 | 1.008 | 0.712 | 5369 | 1162804 |
| bin.1027 | 93.15 | 2.997 | 0.59 | 15758 | 2355063 |
| bin.1028 | 85.13 | 1.539 | 0.452 | 31227 | 1529762 |
| bin.1029 | 68.58 | 4.385 | 0.504 | 7343 | 1455374 |
| bin.103 | 96.79 | 0.671 | 0.517 | 22091 | 2143603 |
| bin.1030 | 77.11 | 4.026 | 0.262 | 12900 | 1354558 |
| bin.1031 | 55.18 | 1.754 | 0.295 | 14524 | 790584 |
| bin.1032 | 89.25 | 3.809 | 0.498 | 24325 | 2545385 |
| bin.1033 | 95.73 | 2.173 | 0.387 | 12406 | 1612630 |
| bin.1034 | 84.79 | 3.655 | 0.567 | 13040 | 1904179 |
| bin.1035 | 78.56 | 0 | 0.497 | 47202 | 1574309 |
| bin.1036 | 97.94 | 1.286 | 0.508 | 28967 | 4405612 |
| bin.1037 | 60.17 | 1.754 | 0.236 | 9495 | 793024 |
| bin.1038 | 86.51 | 2.034 | 0.597 | 6697 | 1735793 |
| bin.1039 | 89.22 | 5.084 | 0.503 | 9881 | 2593590 |
| bin.104 | 94.09 | 3.063 | 0.503 | 13820 | 3063832 |
| bin.1040 | 89.15 | 0.316 | 0.506 | 33717 | 2533126 |
| bin.1041 | 88.23 | 1.612 | 0.518 | 11522 | 1718414 |
| bin.1042 | 52.75 | 0 | 0.399 | 10677 | 1906186 |
| bin.1043 | 89.14 | 3.703 | 0.396 | 22671 | 1696486 |
| bin.1044 | 70.34 | 6.25 | 0.624 | 6275 | 1471744 |
| bin.1045 | 78.87 | 0.671 | 0.291 | 27843 | 1273714 |
| bin.1046 | 79.77 | 4.494 | 0.316 | 6733 | 1109711 |
| bin.1047 | 66.41 | 6.854 | 0.663 | 3871 | 1793923 |
| bin.1048 | 80.92 | 3.061 | 0.635 | 17755 | 2167293 |
| bin.1049 | 81.64 | 5.73 | 0.299 | 7327 | 1166003 |
| bin.105 | 85.07 | 2.896 | 0.348 | 7190 | 1137809 |
| bin.1050 | 94.85 | 0 | 0.485 | 34532 | 2871233 |
| bin.1051 | 90.8 | 0.335 | 0.531 | 18139 | 1597848 |
| bin.1052 | 96.27 | 1.182 | 0.49 | 26115 | 2320096 |
| bin.1053 | 81.88 | 0.34 | 0.518 | 82472 | 1411435 |
| bin.1054 | 72.47 | 7.928 | 0.62 | 4505 | 1882066 |
| bin.1055 | 94.73 | 1.235 | 0.64 | 15666 | 1909502 |
| bin.1056 | 63.79 | 5.172 | 0.592 | 13718 | 1682392 |
| bin.1057 | 65.89 | 0.167 | 0.598 | 17900 | 1424333 |
| bin.1058 | 75.12 | 3.137 | 0.558 | 4343 | 1448311 |
| bin.1059 | 81.25 | 5.113 | 0.487 | 5637 | 2104174 |
| bin.106 | 60.58 | 0.997 | 0.283 | 9624 | 776699 |
| bin.1060 | 78.93 | 0 | 0.264 | 25035 | 992942 |
| bin.1061 | 99.47 | 1.308 | 0.376 | 77640 | 2330644 |
| bin.1062 | 76.69 | 9.389 | 0.63 | 4711 | 1822631 |
| bin.1063 | 96.2 | 1.265 | 0.395 | 26868 | 2472001 |
| bin.1064 | 91.53 | 2.141 | 0.523 | 26469 | 1835519 |
| bin.1065 | 90.05 | 4.362 | 0.552 | 7197 | 1871653 |
| bin.1066 | 84.66 | 2.983 | 0.572 | 11991 | 1637376 |
| bin.1067 | 97.31 | 0 | 0.382 | 33817 | 2836156 |
| bin.1068 | 95.08 | 1.476 | 0.559 | 13993 | 3105994 |
| bin.1069 | 98.3 | 8.065 | 0.498 | 37476 | 1837784 |
| bin.107 | 96.62 | 0 | 0.324 | 11830 | 1532440 |
| bin.1070 | 80.08 | 1.107 | 0.617 | 17397 | 2573779 |
| bin.1071 | 99.16 | 0 | 0.448 | 59062 | 2330343 |
| bin.1072 | 68.59 | 2.3 | 0.315 | 8587 | 1157478 |
| bin.1073 | 94.33 | 1.257 | 0.48 | 15347 | 1904732 |
| bin.1074 | 74.64 | 0.632 | 0.585 | 34784 | 2048209 |
| bin.1075 | 90.36 | 9.294 | 0.585 | 58887 | 2389049 |
| bin.1076 | 52.01 | 9.282 | 0.584 | 6619 | 1971706 |
| bin.1077 | 89.8 | 1.978 | 0.633 | 10072 | 2117337 |
| bin.1078 | 93.75 | 2.572 | 0.391 | 14564 | 2298068 |
| bin.1079 | 79.42 | 0.167 | 0.497 | 12697 | 1588092 |
| bin.108 | 98.27 | 0.919 | 0.35 | 46442 | 2603614 |
| bin.1080 | 97.31 | 2.684 | 0.554 | 51744 | 1736454 |
| bin.1081 | 69.63 | 4.53 | 0.619 | 4231 | 1735748 |
| bin.1082 | 85.17 | 0.721 | 0.523 | 87606 | 2265824 |
| bin.1083 | 90 | 5.369 | 0.545 | 11926 | 2606349 |
| bin.1084 | 76.38 | 2.231 | 0.341 | 9796 | 1384402 |
| bin.1085 | 83.36 | 2.822 | 0.611 | 14188 | 1679054 |
| bin.1086 | 72.15 | 0.238 | 0.544 | 12200 | 1304966 |
| bin.1087 | 79.12 | 1.898 | 0.531 | 8455 | 2056799 |
| bin.1088 | 52.8 | 2.631 | 0.433 | 4793 | 1837447 |
| bin.1089 | 94 | 1.165 | 0.528 | 24088 | 3359917 |
| bin.109 | 59.07 | 8.907 | 0.411 | 5079 | 776007 |
| bin.1090 | 89.24 | 4.09 | 0.527 | 7334 | 2885349 |
| bin.1091 | 73.44 | 1.724 | 0.483 | 18471 | 1645047 |
| bin.1092 | 90 | 1.754 | 0.483 | 35277 | 2136532 |
| bin.1093 | 81.03 | 4.603 | 0.494 | 9047 | 1719272 |
| bin.1094 | 96.98 | 0.714 | 0.5 | 31213 | 1885001 |
| bin.1095 | 62.55 | 5.257 | 0.578 | 3054 | 1533508 |
| bin.1096 | 93.6 | 0.559 | 0.643 | 40189 | 2281816 |
| bin.1097 | 50.8 | 0 | 0.507 | 7684 | 886611 |
| bin.1098 | 77.75 | 0 | 0.511 | 35102 | 2466633 |
| bin.1099 | 92.56 | 4.99 | 0.524 | 11044 | 1465200 |
| bin.11 | 93.02 | 5.369 | 0.583 | 10896 | 2123661 |
| bin.110 | 88.93 | 2.571 | 0.525 | 15396 | 2239620 |
| bin.1100 | 84.21 | 9.389 | 0.681 | 9978 | 1681908 |
| bin.1101 | 55.06 | 6.191 | 0.644 | 3628 | 1503482 |
| bin.1102 | 89.43 | 0.704 | 0.269 | 33677 | 1386241 |
| bin.1103 | 99.13 | 1.486 | 0.462 | 106450 | 4473844 |
| bin.1104 | 61.31 | 3.448 | 0.509 | 7557 | 1939210 |
| bin.1105 | 68.79 | 0 | 0.295 | 4757 | 867084 |
| bin.1106 | 91.51 | 0.671 | 0.521 | 17166 | 2311490 |
| bin.1107 | 62.06 | 2.586 | 0.624 | 26451 | 1595487 |
| bin.1108 | 79.72 | 1.098 | 0.413 | 33854 | 795466 |
| bin.1109 | 74.88 | 2.173 | 0.408 | 4370 | 2151784 |
| bin.111 | 64.81 | 6.896 | 0.465 | 7292 | 1923886 |
| bin.1110 | 78.65 | 5.846 | 0.622 | 5719 | 1567325 |
| bin.1111 | 79.02 | 3.175 | 0.592 | 6334 | 1305252 |
| bin.1112 | 75.67 | 7.214 | 0.622 | 10431 | 2234799 |
| bin.1113 | 72.71 | 0 | 0.453 | 23296 | 626728 |
| bin.1114 | 87.64 | 2.481 | 0.494 | 20251 | 2353886 |
| bin.1115 | 69.83 | 0.37 | 0.516 | 62209 | 2268138 |
| bin.1116 | 86.38 | 2.908 | 0.575 | 5615 | 1552924 |
| bin.1117 | 94.4 | 5.6 | 0.302 | 7066 | 1474779 |
| bin.1118 | 91.86 | 0.455 | 0.564 | 12325 | 1515466 |
| bin.1119 | 62.28 | 0 | 0.3 | 15401 | 996459 |
| bin.112 | 74.77 | 9.239 | 0.506 | 6259 | 1331727 |
| bin.1120 | 75.95 | 1.7 | 0.603 | 12046 | 1797555 |
| bin.1121 | 80.07 | 2.684 | 0.41 | 5103 | 1780541 |
| bin.1122 | 99.2 | 0.039 | 0.526 | 88874 | 3327358 |
| bin.1123 | 61.83 | 4.076 | 0.546 | 10614 | 2210209 |
| bin.1124 | 91.92 | 4.669 | 0.609 | 9977 | 2584631 |
| bin.1125 | 86.45 | 0.561 | 0.298 | 24972 | 1151785 |
| bin.1126 | 56.4 | 0 | 0.464 | 12728 | 2271045 |
| bin.1127 | 83.52 | 2.348 | 0.537 | 4746 | 1714883 |
| bin.1128 | 88.64 | 2.471 | 0.538 | 10310 | 1814413 |
| bin.1129 | 94.76 | 0.514 | 0.473 | 29428 | 2499537 |
| bin.113 | 67.74 | 4.31 | 0.549 | 5595 | 2156101 |
| bin.1130 | 97.2 | 0 | 0.404 | 89744 | 2583164 |
| bin.1131 | 86.65 | 2.619 | 0.464 | 32206 | 2101270 |
| bin.1132 | 71.52 | 5.664 | 0.269 | 11914 | 1161256 |
| bin.1133 | 96.36 | 0.187 | 0.39 | 75897 | 2708053 |
| bin.1134 | 58.8 | 3.243 | 0.265 | 11156 | 988806 |
| bin.1135 | 71.28 | 8.952 | 0.505 | 6415 | 1933494 |
| bin.1136 | 75.08 | 1.098 | 0.427 | 12950 | 858129 |
| bin.1137 | 81.93 | 5.454 | 0.279 | 12420 | 1055711 |
| bin.1138 | 72.54 | 10 | 0.627 | 5387 | 1915008 |
| bin.1139 | 50 | 5.263 | 0.267 | 3712 | 919399 |
| bin.114 | 59.96 | 1.172 | 0.537 | 17475 | 1908613 |
| bin.1141 | 50 | 0 | 0.624 | 14036 | 772916 |
| bin.1142 | 50 | 5.172 | 0.566 | 3414 | 881546 |
| bin.1143 | 50 | 3.571 | 0.661 | 6334 | 1335911 |
| bin.115 | 92.44 | 2.572 | 0.468 | 8879 | 1851005 |
| bin.116 | 86.33 | 4.503 | 0.631 | 13575 | 2453121 |
| bin.117 | 52.74 | 0 | 0.3 | 5314 | 2503763 |
| bin.118 | 55.02 | 1.754 | 0.285 | 6701 | 933800 |
| bin.119 | 77.25 | 2.112 | 0.383 | 4175 | 1086049 |
| bin.12 | 68.46 | 1.698 | 0.285 | 49926 | 1525355 |
| bin.120 | 84.73 | 2.069 | 0.508 | 14381 | 1405562 |
| bin.121 | 67.14 | 0 | 0.61 | 10214 | 960662 |
| bin.122 | 74.3 | 0.641 | 0.477 | 14209 | 1592966 |
| bin.123 | 60.9 | 2.125 | 0.605 | 5828 | 1762037 |
| bin.124 | 99.04 | 1.725 | 0.389 | 14307 | 1368293 |
| bin.125 | 77.19 | 3.97 | 0.626 | 7011 | 1723425 |
| bin.126 | 55.94 | 0 | 0.445 | 3769 | 1186641 |
| bin.127 | 54.3 | 4.069 | 0.546 | 23140 | 1761611 |
| bin.128 | 93.66 | 2.083 | 0.489 | 34887 | 1386368 |
| bin.129 | 79.8 | 3.489 | 0.519 | 4862 | 1910097 |
| bin.13 | 73 | 1.898 | 0.575 | 10886 | 1465362 |
| bin.130 | 83.69 | 1.25 | 0.546 | 9111 | 1378845 |
| bin.131 | 90.89 | 3.942 | 0.593 | 102325 | 2717138 |
| bin.132 | 79.12 | 4.075 | 0.555 | 5361 | 2512663 |
| bin.133 | 74.52 | 3.208 | 0.515 | 6910 | 1565331 |
| bin.134 | 53.6 | 4.31 | 0.68 | 5805 | 1387273 |
| bin.135 | 94.18 | 0.223 | 0.375 | 127697 | 1777515 |
| bin.136 | 97.12 | 1.685 | 0.454 | 81381 | 3168098 |
| bin.137 | 69.13 | 1.748 | 0.261 | 13080 | 1200510 |
| bin.138 | 83.34 | 4.052 | 0.463 | 5385 | 1761417 |
| bin.139 | 72.58 | 2.236 | 0.263 | 9845 | 1403977 |
| bin.14 | 95.23 | 0.952 | 0.433 | 18373 | 1609040 |
| bin.140 | 94.5 | 3.195 | 0.634 | 8104 | 2677616 |
| bin.141 | 89.64 | 3.688 | 0.354 | 7242 | 2172737 |
| bin.142 | 99.04 | 2.857 | 0.53 | 41466 | 1994164 |
| bin.143 | 89.37 | 0.671 | 0.435 | 11311 | 1703498 |
| bin.144 | 71.38 | 1.685 | 0.266 | 9887 | 835913 |
| bin.145 | 92.45 | 2.906 | 0.301 | 30906 | 1161969 |
| bin.146 | 70.61 | 7.791 | 0.53 | 4192 | 2714047 |
| bin.147 | 81.61 | 1.435 | 0.468 | 9534 | 2232338 |
| bin.148 | 99.05 | 0.943 | 0.442 | 30916 | 1978314 |
| bin.149 | 78.64 | 4.508 | 0.622 | 6695 | 1769967 |
| bin.15 | 72.02 | 3.807 | 0.349 | 20676 | 1580951 |
| bin.150 | 95.97 | 1.384 | 0.487 | 40222 | 2059273 |
| bin.151 | 86.86 | 1.034 | 0.623 | 10567 | 1540035 |
| bin.152 | 98.36 | 0 | 0.391 | 31500 | 1478780 |
| bin.153 | 87.22 | 0 | 0.46 | 182673 | 1631206 |
| bin.154 | 59.78 | 5.537 | 0.603 | 4409 | 1729214 |
| bin.155 | 78.3 | 0.476 | 0.521 | 28103 | 1965886 |
| bin.156 | 90.15 | 0.847 | 0.492 | 34685 | 2676072 |
| bin.157 | 96.14 | 4.097 | 0.445 | 17671 | 4586050 |
| bin.158 | 95.96 | 0.806 | 0.436 | 71801 | 1217179 |
| bin.159 | 52.58 | 1.724 | 0.637 | 5147 | 1630279 |
| bin.16 | 76.29 | 0.292 | 0.547 | 6482 | 1646037 |
| bin.160 | 63.25 | 0 | 0.482 | 27501 | 1101311 |
| bin.161 | 86.72 | 1.139 | 0.554 | 10529 | 1704939 |
| bin.162 | 96.27 | 0.692 | 0.439 | 21307 | 2937494 |
| bin.163 | 50.74 | 0 | 0.252 | 13082 | 1032232 |
| bin.164 | 85.58 | 0.099 | 0.37 | 38106 | 874060 |
| bin.165 | 86.57 | 1.677 | 0.484 | 16342 | 2346615 |
| bin.166 | 78.73 | 1.901 | 0.572 | 9838 | 2139834 |
| bin.167 | 57.97 | 0.478 | 0.493 | 7801 | 1452249 |
| bin.168 | 80.19 | 4.809 | 0.643 | 7889 | 1898414 |
| bin.169 | 87.23 | 0.945 | 0.477 | 15202 | 1988264 |
| bin.17 | 50.31 | 0 | 0.498 | 7915 | 1209168 |
| bin.170 | 92.92 | 5.298 | 0.493 | 18598 | 1586954 |
| bin.171 | 79.97 | 2.969 | 0.336 | 6991 | 989282 |
| bin.172 | 96.8 | 0 | 0.482 | 25282 | 1716767 |
| bin.173 | 88.94 | 2.241 | 0.49 | 15609 | 2659816 |
| bin.174 | 85.25 | 0.843 | 0.469 | 30924 | 2629737 |
| bin.175 | 61.59 | 4.814 | 0.53 | 18302 | 2128493 |
| bin.176 | 59.34 | 7.017 | 0.448 | 5174 | 2026631 |
| bin.177 | 66.29 | 6.14 | 0.607 | 12017 | 2166781 |
| bin.178 | 96.64 | 0.335 | 0.485 | 28722 | 2195000 |
| bin.179 | 98.65 | 0.838 | 0.391 | 83274 | 1807993 |
| bin.18 | 79.27 | 0 | 0.306 | 24932 | 1128168 |
| bin.180 | 83.01 | 4.178 | 0.511 | 7568 | 1601621 |
| bin.181 | 84.26 | 0.16 | 0.262 | 17972 | 990664 |
| bin.182 | 95.95 | 1.746 | 0.493 | 52193 | 2374220 |
| bin.183 | 66.73 | 3.579 | 0.266 | 18488 | 1351854 |
| bin.184 | 97.57 | 2.348 | 0.613 | 26958 | 1780499 |
| bin.185 | 57 | 0 | 0.453 | 10202 | 1537676 |
| bin.186 | 63.78 | 2.181 | 0.334 | 5384 | 1077758 |
| bin.187 | 87.56 | 0.099 | 0.513 | 12191 | 1516362 |
| bin.188 | 55.52 | 0.968 | 0.533 | 10006 | 1079325 |
| bin.189 | 91.12 | 4.166 | 0.58 | 25373 | 1734502 |
| bin.19 | 81.53 | 8.562 | 0.589 | 7754 | 2023007 |
| bin.190 | 100 | 0 | 0.406 | 185273 | 3504735 |
| bin.191 | 93.42 | 1.582 | 0.482 | 29610 | 3126789 |
| bin.192 | 81.07 | 4.362 | 0.503 | 6843 | 1602282 |
| bin.193 | 79.86 | 2.597 | 0.458 | 10431 | 826242 |
| bin.194 | 52.87 | 3.448 | 0.618 | 4596 | 1578171 |
| bin.195 | 86.94 | 2.808 | 0.271 | 7967 | 941336 |
| bin.196 | 92.87 | 1.446 | 0.534 | 21974 | 2047550 |
| bin.197 | 54.59 | 1.647 | 0.526 | 3588 | 1205837 |
| bin.198 | 67.96 | 8.724 | 0.451 | 6041 | 2826243 |
| bin.199 | 87.92 | 1.685 | 0.289 | 17130 | 1025576 |
| bin.2 | 89.28 | 3.605 | 0.426 | 14395 | 2331683 |
| bin.20 | 83.57 | 0.943 | 0.467 | 35896 | 1760569 |
| bin.200 | 95.71 | 0 | 0.507 | 145557 | 1765169 |
| bin.201 | 53.28 | 2.419 | 0.503 | 8097 | 1454799 |
| bin.202 | 78.08 | 0.16 | 0.26 | 14314 | 961110 |
| bin.203 | 73.19 | 1.148 | 0.481 | 9779 | 1815466 |
| bin.204 | 70.99 | 2.534 | 0.555 | 7536 | 1707271 |
| bin.205 | 92.13 | 3.018 | 0.328 | 24333 | 1207286 |
| bin.206 | 96.82 | 0.634 | 0.494 | 58268 | 2359714 |
| bin.207 | 84.66 | 3.007 | 0.474 | 29409 | 2112600 |
| bin.208 | 76.85 | 3.37 | 0.281 | 10268 | 1183449 |
| bin.209 | 53.19 | 0 | 0.59 | 3218 | 896611 |
| bin.21 | 88 | 6.856 | 0.531 | 6051 | 2834464 |
| bin.210 | 58.08 | 3.448 | 0.579 | 27670 | 2336056 |
| bin.211 | 97.61 | 0 | 0.56 | 65527 | 2594757 |
| bin.212 | 95.53 | 2.595 | 0.537 | 13883 | 2033791 |
| bin.213 | 67.36 | 1.546 | 0.264 | 8959 | 1399953 |
| bin.214 | 62.51 | 4.301 | 0.264 | 11693 | 1022169 |
| bin.215 | 54.95 | 7.471 | 0.566 | 2518 | 1353251 |
| bin.216 | 92.57 | 1.773 | 0.48 | 17015 | 1813962 |
| bin.217 | 85.08 | 0.335 | 0.473 | 5868 | 1416896 |
| bin.218 | 91.82 | 3.733 | 0.505 | 8833 | 2304766 |
| bin.219 | 81.5 | 3.803 | 0.496 | 11898 | 1959042 |
| bin.22 | 88.45 | 6.544 | 0.596 | 7128 | 2287259 |
| bin.220 | 68.39 | 0 | 0.529 | 26103 | 1282679 |
| bin.221 | 80.21 | 1.906 | 0.712 | 16430 | 1673263 |
| bin.222 | 67.81 | 5.185 | 0.52 | 4364 | 1890656 |
| bin.223 | 89.87 | 1.465 | 0.546 | 13849 | 2291015 |
| bin.224 | 64.19 | 0 | 0.372 | 4051 | 1531536 |
| bin.225 | 62.26 | 2.294 | 0.364 | 5022 | 872029 |
| bin.226 | 73.03 | 0.502 | 0.526 | 5783 | 1484304 |
| bin.227 | 86.29 | 8.587 | 0.251 | 5388 | 1061993 |
| bin.228 | 59.07 | 9.183 | 0.66 | 4160 | 1611178 |
| bin.229 | 70.84 | 0 | 0.598 | 37329 | 2912239 |
| bin.23 | 69.82 | 2.631 | 0.294 | 8326 | 1253988 |
| bin.230 | 99.62 | 0.566 | 0.429 | 84567 | 2718364 |
| bin.231 | 95.8 | 0 | 0.451 | 39295 | 2008045 |
| bin.232 | 63.13 | 0.447 | 0.613 | 9517 | 1528079 |
| bin.233 | 65.61 | 1.048 | 0.268 | 11521 | 1055077 |
| bin.234 | 91.25 | 7.258 | 0.695 | 8741 | 2135993 |
| bin.235 | 78.87 | 7.795 | 0.606 | 5177 | 1868383 |
| bin.236 | 66.39 | 1.364 | 0.264 | 12136 | 1017009 |
| bin.237 | 96.55 | 3.257 | 0.346 | 13593 | 1503142 |
| bin.238 | 98.46 | 0.951 | 0.462 | 28718 | 1788550 |
| bin.239 | 88.57 | 4.647 | 0.614 | 7040 | 2234139 |
| bin.24 | 60.65 | 1.724 | 0.53 | 14117 | 1490702 |
| bin.240 | 95.73 | 0 | 0.557 | 30114 | 3072672 |
| bin.241 | 94.81 | 0.042 | 0.328 | 20908 | 2177548 |
| bin.242 | 86.46 | 0.227 | 0.529 | 14788 | 1719177 |
| bin.243 | 95.07 | 4.25 | 0.42 | 25926 | 2252061 |
| bin.244 | 90.93 | 0.838 | 0.469 | 41903 | 2450822 |
| bin.245 | 83.27 | 3.666 | 0.635 | 29898 | 1498604 |
| bin.246 | 59.98 | 3.448 | 0.585 | 7064 | 1660296 |
| bin.247 | 85.44 | 2.572 | 0.404 | 5563 | 1859866 |
| bin.248 | 67.18 | 0 | 0.302 | 7567 | 992248 |
| bin.249 | 70.54 | 1.442 | 0.28 | 22918 | 1292414 |
| bin.25 | 81.03 | 3.448 | 0.265 | 12544 | 4148115 |
| bin.250 | 54.71 | 3.636 | 0.291 | 5881 | 1087690 |
| bin.251 | 86.52 | 1.342 | 0.48 | 7280 | 1757476 |
| bin.252 | 97.97 | 0.566 | 0.586 | 130662 | 2566729 |
| bin.253 | 82.91 | 2.098 | 0.478 | 6883 | 2044933 |
| bin.254 | 69.71 | 3.187 | 0.265 | 19875 | 1430237 |
| bin.255 | 94.47 | 3.145 | 0.567 | 14722 | 2357358 |
| bin.256 | 76.91 | 2.445 | 0.65 | 14498 | 1795074 |
| bin.257 | 79.51 | 2.237 | 0.51 | 5938 | 1446326 |
| bin.258 | 79.16 | 2.042 | 0.558 | 9235 | 1257791 |
| bin.259 | 90.91 | 1.886 | 0.281 | 15215 | 2304449 |
| bin.26 | 95.16 | 1.209 | 0.487 | 22133 | 2089256 |
| bin.260 | 94.16 | 2.043 | 0.569 | 63359 | 2313222 |
| bin.261 | 79.5 | 5.538 | 0.643 | 3634 | 1876984 |
| bin.262 | 55.3 | 0 | 0.65 | 4568 | 1648568 |
| bin.263 | 99.08 | 2.004 | 0.591 | 12530 | 2420183 |
| bin.264 | 77.24 | 0.644 | 0.423 | 117644 | 2542712 |
| bin.265 | 55.78 | 3.448 | 0.277 | 3796 | 1111258 |
| bin.266 | 89.98 | 3.37 | 0.29 | 37027 | 1831341 |
| bin.267 | 100 | 0.24 | 0.581 | 179949 | 2607931 |
| bin.268 | 95.9 | 0.584 | 0.355 | 69310 | 1948253 |
| bin.269 | 66.37 | 0.917 | 0.294 | 11374 | 1140685 |
| bin.27 | 79.78 | 7.465 | 0.562 | 5668 | 2315430 |
| bin.270 | 99.05 | 1.886 | 0.35 | 16977 | 2124955 |
| bin.271 | 94.33 | 1.415 | 0.537 | 12262 | 1868389 |
| bin.272 | 94.22 | 2.01 | 0.475 | 35301 | 1639873 |
| bin.273 | 78.77 | 2.125 | 0.641 | 10559 | 2014992 |
| bin.274 | 90.55 | 1.269 | 0.536 | 162366 | 2362632 |
| bin.275 | 84.41 | 0 | 0.432 | 28521 | 794715 |
| bin.276 | 97.25 | 0.223 | 0.404 | 38719 | 1891199 |
| bin.277 | 60.12 | 1.123 | 0.282 | 3149 | 840258 |
| bin.278 | 82.23 | 6.056 | 0.294 | 5713 | 1378951 |
| bin.279 | 63.79 | 2.586 | 0.692 | 9024 | 1278090 |
| bin.28 | 71.63 | 1.612 | 0.347 | 10232 | 1027478 |
| bin.280 | 70.58 | 1.291 | 0.522 | 8620 | 1446943 |
| bin.281 | 86.58 | 1.424 | 0.356 | 16658 | 1686473 |
| bin.282 | 62.38 | 4.31 | 0.513 | 4628 | 2032107 |
| bin.283 | 85.25 | 3.001 | 0.415 | 12083 | 1675766 |
| bin.284 | 71.88 | 3.624 | 0.3 | 11162 | 1242834 |
| bin.285 | 91.16 | 2.181 | 0.465 | 26426 | 1787193 |
| bin.286 | 81.88 | 1.509 | 0.477 | 23679 | 2232337 |
| bin.287 | 73.25 | 1.685 | 0.319 | 5188 | 1016285 |
| bin.288 | 69.13 | 1.953 | 0.342 | 3056 | 1062883 |
| bin.289 | 50.9 | 3.733 | 0.353 | 4229 | 737048 |
| bin.29 | 72.41 | 6.393 | 0.499 | 6014 | 2063004 |
| bin.290 | 86.89 | 4.273 | 0.333 | 130557 | 1958258 |
| bin.291 | 80.1 | 8.199 | 0.554 | 4274 | 2251807 |
| bin.292 | 93.06 | 1.551 | 0.432 | 37080 | 2278296 |
| bin.293 | 97.75 | 0 | 0.264 | 25188 | 1501857 |
| bin.294 | 90.07 | 9.543 | 0.595 | 11153 | 2821083 |
| bin.295 | 89.82 | 5.503 | 0.347 | 7015 | 2264287 |
| bin.296 | 92.06 | 0.806 | 0.487 | 35063 | 1532441 |
| bin.297 | 53.8 | 0 | 0.28 | 7728 | 712995 |
| bin.298 | 52.55 | 8.771 | 0.271 | 6469 | 742944 |
| bin.299 | 76.85 | 4.08 | 0.32 | 4832 | 1983912 |
| bin.3 | 61.67 | 2.339 | 0.541 | 7647 | 1308379 |
| bin.30 | 70.72 | 2.531 | 0.485 | 6940 | 1483949 |
| bin.300 | 78.76 | 0.961 | 0.568 | 5264 | 1424104 |
| bin.301 | 61.91 | 1.754 | 0.458 | 4408 | 1866023 |
| bin.302 | 86.87 | 1.118 | 0.534 | 14019 | 2102099 |
| bin.303 | 56.69 | 3.448 | 0.625 | 7304 | 1786710 |
| bin.304 | 95.97 | 0 | 0.432 | 74704 | 1713887 |
| bin.305 | 54.91 | 8.862 | 0.587 | 5516 | 2014362 |
| bin.306 | 73.88 | 1.677 | 0.614 | 4286 | 1200828 |
| bin.307 | 70.45 | 9.739 | 0.491 | 5449 | 1760855 |
| bin.308 | 88.75 | 0 | 0.588 | 31151 | 1384287 |
| bin.309 | 76.17 | 0 | 0.267 | 9814 | 938690 |
| bin.31 | 92.45 | 3.301 | 0.294 | 11823 | 2542489 |
| bin.310 | 92.86 | 1.793 | 0.482 | 12395 | 2718900 |
| bin.311 | 83.42 | 7.491 | 0.42 | 12466 | 1820412 |
| bin.312 | 77.89 | 1.098 | 0.409 | 13073 | 861235 |
| bin.313 | 81.78 | 0.476 | 0.507 | 6866 | 1543632 |
| bin.314 | 92.37 | 1.118 | 0.52 | 19002 | 2097607 |
| bin.315 | 79.36 | 2.016 | 0.505 | 5127 | 1192190 |
| bin.316 | 72.41 | 5.617 | 0.27 | 4762 | 1026696 |
| bin.317 | 76.89 | 2.419 | 0.593 | 10350 | 2287228 |
| bin.318 | 81.69 | 2.969 | 0.281 | 10826 | 1137612 |
| bin.319 | 72.54 | 2.808 | 0.295 | 6970 | 857899 |
| bin.32 | 93.19 | 3.436 | 0.351 | 8142 | 1527874 |
| bin.320 | 82.85 | 0.48 | 0.532 | 57603 | 1465706 |
| bin.321 | 99.46 | 0 | 0.433 | 129214 | 4288547 |
| bin.322 | 97.11 | 9.497 | 0.624 | 27868 | 3087171 |
| bin.323 | 72.13 | 0.972 | 0.485 | 24867 | 2409268 |
| bin.324 | 59.92 | 6.41 | 0.259 | 9236 | 1239603 |
| bin.325 | 69.87 | 0.073 | 0.502 | 4489 | 1143093 |
| bin.326 | 93.95 | 0.806 | 0.492 | 43062 | 1356416 |
| bin.327 | 93.98 | 0.671 | 0.488 | 37891 | 2078413 |
| bin.328 | 78.52 | 1.724 | 0.315 | 16771 | 1755663 |
| bin.329 | 65.09 | 7.067 | 0.581 | 6281 | 1365852 |
| bin.33 | 67.84 | 2.631 | 0.311 | 9689 | 1108062 |
| bin.330 | 82.59 | 7.455 | 0.518 | 4847 | 1502218 |
| bin.331 | 85.06 | 6.655 | 0.405 | 4627 | 1859377 |
| bin.332 | 97.65 | 0 | 0.467 | 20746 | 2708282 |
| bin.333 | 72.21 | 1.869 | 0.602 | 4818 | 1103169 |
| bin.334 | 83.97 | 3.733 | 0.647 | 13170 | 1989600 |
| bin.335 | 76.19 | 1.57 | 0.429 | 9127 | 1177812 |
| bin.336 | 96.77 | 3.225 | 0.362 | 30484 | 1723635 |
| bin.337 | 84.91 | 4.809 | 0.603 | 9219 | 1507972 |
| bin.338 | 74.31 | 0.02 | 0.263 | 33329 | 1195836 |
| bin.339 | 83.51 | 8.341 | 0.444 | 25796 | 902050 |
| bin.34 | 53.74 | 2.016 | 0.646 | 4868 | 1232870 |
| bin.340 | 85.63 | 1.804 | 0.483 | 10077 | 2563744 |
| bin.341 | 88.14 | 3.243 | 0.552 | 8175 | 2783909 |
| bin.342 | 63.29 | 1.189 | 0.508 | 5934 | 1800392 |
| bin.343 | 99.24 | 1.32 | 0.389 | 85920 | 3427906 |
| bin.344 | 94.35 | 4.029 | 0.515 | 7188 | 1525825 |
| bin.345 | 56.09 | 1.333 | 0.273 | 5894 | 921505 |
| bin.346 | 91.89 | 1.686 | 0.594 | 6826 | 1984719 |
| bin.347 | 65.88 | 6.979 | 0.525 | 2804 | 1872902 |
| bin.348 | 89.89 | 2.905 | 0.36 | 10784 | 2790694 |
| bin.349 | 93.9 | 1.19 | 0.597 | 18302 | 1925288 |
| bin.35 | 56.54 | 3.159 | 0.533 | 22942 | 1674588 |
| bin.350 | 90.95 | 2.674 | 0.464 | 24416 | 2763025 |
| bin.351 | 85.39 | 6.741 | 0.247 | 14219 | 1154208 |
| bin.352 | 78.09 | 1.282 | 0.6 | 9235 | 1998095 |
| bin.353 | 97.16 | 1.273 | 0.446 | 31635 | 2735698 |
| bin.354 | 53.22 | 0.671 | 0.494 | 9104 | 990793 |
| bin.355 | 88.39 | 0 | 0.573 | 6087 | 969269 |
| bin.356 | 59.48 | 1.724 | 0.652 | 5876 | 1484234 |
| bin.357 | 98.71 | 1.51 | 0.55 | 78422 | 3170783 |
| bin.358 | 50.97 | 0 | 0.468 | 8220 | 1035158 |
| bin.359 | 65.77 | 0.223 | 0.608 | 12234 | 1437204 |
| bin.36 | 71.11 | 1.428 | 0.402 | 5400 | 944424 |
| bin.360 | 70.73 | 1.565 | 0.484 | 4072 | 1499674 |
| bin.361 | 57.38 | 5.172 | 0.635 | 5547 | 1486768 |
| bin.362 | 88.98 | 2.237 | 0.489 | 14234 | 1621307 |
| bin.363 | 61.36 | 8.62 | 0.637 | 4617 | 1618376 |
| bin.364 | 80.91 | 2.012 | 0.486 | 13567 | 2287542 |
| bin.365 | 58.93 | 1.123 | 0.459 | 2745 | 738701 |
| bin.366 | 83.63 | 4.324 | 0.516 | 10531 | 2807176 |
| bin.367 | 95.8 | 0.671 | 0.528 | 55192 | 1723206 |
| bin.368 | 94.98 | 4.53 | 0.455 | 53141 | 2506090 |
| bin.369 | 50.86 | 0 | 0.449 | 10076 | 2195608 |
| bin.37 | 90.44 | 2.109 | 0.508 | 21813 | 2935655 |
| bin.370 | 97.31 | 0 | 0.429 | 58500 | 2061839 |
| bin.371 | 74.63 | 5.497 | 0.523 | 5093 | 1802340 |
| bin.372 | 77.89 | 3.26 | 0.526 | 5944 | 1884941 |
| bin.373 | 88.03 | 0.671 | 0.58 | 18611 | 2432394 |
| bin.374 | 84.19 | 2.564 | 0.415 | 7544 | 1787919 |
| bin.375 | 60.3 | 1.686 | 0.52 | 4286 | 1048247 |
| bin.376 | 74.98 | 6.329 | 0.55 | 5423 | 2418453 |
| bin.377 | 83.66 | 3.467 | 0.637 | 6826 | 1625109 |
| bin.378 | 51.75 | 5.263 | 0.246 | 4543 | 974177 |
| bin.379 | 67.04 | 9.42 | 0.573 | 5689 | 1761435 |
| bin.38 | 83.25 | 0.862 | 0.537 | 26055 | 3750293 |
| bin.380 | 50.44 | 0 | 0.63 | 5379 | 991335 |
| bin.381 | 97.46 | 3.914 | 0.618 | 24477 | 2199506 |
| bin.382 | 93.45 | 3.481 | 0.587 | 15194 | 2239516 |
| bin.383 | 88.37 | 2.016 | 0.521 | 12830 | 1586131 |
| bin.384 | 90.52 | 5.402 | 0.441 | 11118 | 1941388 |
| bin.385 | 74.72 | 1.465 | 0.372 | 22012 | 779710 |
| bin.386 | 65.21 | 1.048 | 0.274 | 22239 | 1167503 |
| bin.387 | 98.55 | 2.617 | 0.564 | 95293 | 2320949 |
| bin.388 | 92.58 | 0 | 0.299 | 50477 | 1821381 |
| bin.389 | 67.55 | 4.31 | 0.507 | 4076 | 1910855 |
| bin.39 | 90.71 | 7.514 | 0.631 | 19035 | 3115405 |
| bin.390 | 90.61 | 1.342 | 0.345 | 9998 | 2739352 |
| bin.391 | 72.24 | 3.145 | 0.667 | 9706 | 2029760 |
| bin.392 | 56.14 | 6.896 | 0.631 | 8941 | 1664933 |
| bin.393 | 93.42 | 1.398 | 0.437 | 19035 | 1509335 |
| bin.394 | 83.62 | 2.963 | 0.596 | 11167 | 2251470 |
| bin.395 | 83.5 | 1.174 | 0.41 | 8330 | 1505405 |
| bin.396 | 55.62 | 2.146 | 0.269 | 7446 | 919761 |
| bin.397 | 67.85 | 2.069 | 0.322 | 8874 | 1123032 |
| bin.398 | 96.25 | 3.635 | 0.344 | 36909 | 3034676 |
| bin.399 | 95 | 4.559 | 0.351 | 13907 | 2399486 |
| bin.4 | 55.63 | 0 | 0.516 | 14085 | 724835 |
| bin.40 | 98.52 | 2.567 | 0.45 | 21883 | 3616923 |
| bin.400 | 92.39 | 0 | 0.478 | 45172 | 1861075 |
| bin.401 | 97.37 | 2.531 | 0.464 | 35629 | 3018757 |
| bin.402 | 50.84 | 6.536 | 0.603 | 3589 | 1560754 |
| bin.403 | 83.18 | 3.866 | 0.507 | 7556 | 2392275 |
| bin.404 | 70.82 | 1.381 | 0.508 | 7825 | 1208876 |
| bin.405 | 67.68 | 0 | 0.504 | 42138 | 1339414 |
| bin.406 | 72.67 | 7.954 | 0.606 | 8491 | 2424000 |
| bin.407 | 63.73 | 3.12 | 0.652 | 5268 | 1385947 |
| bin.408 | 78.42 | 4.895 | 0.263 | 16621 | 1397892 |
| bin.409 | 99.03 | 1.075 | 0.587 | 31864 | 2823477 |
| bin.41 | 86.03 | 0.854 | 0.294 | 71823 | 1896431 |
| bin.410 | 69.23 | 0.699 | 0.34 | 17138 | 1071550 |
| bin.411 | 74.52 | 0.763 | 0.422 | 13352 | 907163 |
| bin.412 | 70.87 | 1.932 | 0.275 | 19886 | 1273467 |
| bin.413 | 82.04 | 4.761 | 0.476 | 32525 | 2115789 |
| bin.414 | 88.29 | 0 | 0.5 | 16505 | 2254198 |
| bin.415 | 97.31 | 0.146 | 0.591 | 47923 | 1984633 |
| bin.416 | 69.54 | 4.446 | 0.584 | 7388 | 1650915 |
| bin.417 | 60.18 | 0 | 0.273 | 7623 | 750567 |
| bin.418 | 66.72 | 6.896 | 0.544 | 4657 | 1745260 |
| bin.419 | 90.67 | 7.491 | 0.47 | 7272 | 2438697 |
| bin.42 | 83.71 | 3.801 | 0.539 | 5964 | 1305371 |
| bin.420 | 71.22 | 3.523 | 0.276 | 29898 | 1300546 |
| bin.421 | 89.65 | 0.713 | 0.568 | 18843 | 1685215 |
| bin.422 | 66.71 | 2.564 | 0.366 | 6105 | 1511304 |
| bin.423 | 96.42 | 1.363 | 0.459 | 40987 | 2090591 |
| bin.424 | 84.31 | 4.375 | 0.493 | 11891 | 1588974 |
| bin.425 | 72.47 | 3.857 | 0.287 | 4132 | 872846 |
| bin.426 | 75.24 | 4.086 | 0.584 | 42351 | 3011746 |
| bin.427 | 74.07 | 0 | 0.302 | 18442 | 738640 |
| bin.428 | 97.16 | 0 | 0.429 | 47797 | 1769478 |
| bin.429 | 91.29 | 3.961 | 0.491 | 8957 | 2636587 |
| bin.43 | 92.04 | 0 | 0.46 | 56821 | 2122078 |
| bin.430 | 67.79 | 6.605 | 0.373 | 4587 | 1254595 |
| bin.431 | 86.24 | 0 | 0.503 | 22600 | 1409212 |
| bin.432 | 75.15 | 0.838 | 0.514 | 11911 | 1611785 |
| bin.433 | 86.44 | 1.107 | 0.466 | 94548 | 1768175 |
| bin.434 | 56.33 | 0 | 0.279 | 3686 | 746267 |
| bin.435 | 81.82 | 2.354 | 0.54 | 16699 | 2872555 |
| bin.436 | 94 | 4.545 | 0.473 | 10767 | 2379701 |
| bin.437 | 59.42 | 3.508 | 0.256 | 3913 | 948046 |
| bin.438 | 96 | 4.232 | 0.276 | 10724 | 1809491 |
| bin.439 | 58.58 | 0 | 0.585 | 3131 | 1407778 |
| bin.44 | 98.58 | 2.452 | 0.376 | 14087 | 2150210 |
| bin.440 | 77.15 | 2.369 | 0.558 | 6851 | 2276577 |
| bin.441 | 78.88 | 7.495 | 0.503 | 5350 | 1652627 |
| bin.442 | 69.31 | 1.724 | 0.259 | 11338 | 2116845 |
| bin.443 | 82.81 | 6.329 | 0.514 | 8911 | 1702523 |
| bin.444 | 93.84 | 1.738 | 0.624 | 13176 | 1764127 |
| bin.445 | 57.52 | 3.508 | 0.236 | 6445 | 703279 |
| bin.446 | 97.17 | 1.759 | 0.596 | 19757 | 2628928 |
| bin.447 | 78.83 | 6.65 | 0.609 | 12651 | 2041823 |
| bin.448 | 56.53 | 5.172 | 0.698 | 6005 | 1537561 |
| bin.449 | 59.99 | 1.923 | 0.634 | 31478 | 1534042 |
| bin.45 | 66.98 | 3.508 | 0.288 | 7445 | 973053 |
| bin.450 | 58.98 | 5.257 | 0.295 | 6213 | 1122235 |
| bin.451 | 64.23 | 1.048 | 0.276 | 8339 | 886706 |
| bin.452 | 88.36 | 0 | 0.589 | 29477 | 2003379 |
| bin.453 | 97.87 | 0.066 | 0.48 | 42654 | 1912980 |
| bin.454 | 93 | 0.335 | 0.507 | 12608 | 2744417 |
| bin.455 | 93.45 | 0 | 0.469 | 41213 | 2729642 |
| bin.456 | 70.14 | 3.387 | 0.584 | 5103 | 1141699 |
| bin.457 | 67.52 | 6.487 | 0.257 | 11520 | 1518092 |
| bin.458 | 68.27 | 0 | 0.492 | 9286 | 2359328 |
| bin.459 | 96.79 | 0.591 | 0.597 | 89375 | 4243908 |
| bin.46 | 85.93 | 7.303 | 0.314 | 9683 | 1312524 |
| bin.460 | 95.59 | 0.806 | 0.575 | 11877 | 3061112 |
| bin.461 | 86.32 | 1.282 | 0.373 | 119357 | 2020274 |
| bin.462 | 77.28 | 3.565 | 0.552 | 4926 | 1593524 |
| bin.463 | 98.33 | 1.307 | 0.508 | 253302 | 1540951 |
| bin.464 | 79.17 | 9.401 | 0.612 | 5103 | 2181460 |
| bin.465 | 66.72 | 1.724 | 0.555 | 10696 | 2106975 |
| bin.466 | 94.75 | 0.429 | 0.498 | 29116 | 1970146 |
| bin.467 | 84.11 | 2.684 | 0.355 | 6929 | 1744327 |
| bin.468 | 95.76 | 3.355 | 0.496 | 15416 | 2581963 |
| bin.469 | 72.46 | 1.36 | 0.478 | 35328 | 1409627 |
| bin.47 | 80.84 | 0.073 | 0.577 | 15218 | 1254501 |
| bin.470 | 85.08 | 8.602 | 0.681 | 7739 | 3030822 |
| bin.471 | 87.18 | 4.905 | 0.316 | 4962 | 2007058 |
| bin.472 | 51 | 1.398 | 0.279 | 4862 | 855367 |
| bin.473 | 95.96 | 0.476 | 0.511 | 47665 | 2449949 |
| bin.474 | 87.92 | 2.969 | 0.321 | 13110 | 1014913 |
| bin.475 | 58.47 | 4.222 | 0.577 | 3749 | 1651215 |
| bin.476 | 71.53 | 3.988 | 0.339 | 3820 | 1544653 |
| bin.477 | 90.69 | 4.308 | 0.544 | 25042 | 1892925 |
| bin.478 | 92.58 | 3.241 | 0.441 | 56389 | 3042188 |
| bin.479 | 77.95 | 0 | 0.525 | 15221 | 1773472 |
| bin.48 | 95.96 | 3.457 | 0.615 | 25726 | 2733731 |
| bin.480 | 87.32 | 5.136 | 0.578 | 10342 | 2100066 |
| bin.481 | 90.13 | 0.223 | 0.558 | 20795 | 1366213 |
| bin.482 | 88.83 | 4.194 | 0.522 | 6214 | 2251640 |
| bin.483 | 96 | 0.533 | 0.275 | 86660 | 3155801 |
| bin.484 | 55.07 | 0.139 | 0.569 | 5878 | 1041447 |
| bin.485 | 57.69 | 3.448 | 0.41 | 4928 | 2084121 |
| bin.486 | 97.71 | 5.204 | 0.435 | 73776 | 3478644 |
| bin.487 | 85.64 | 0 | 0.569 | 122681 | 2344759 |
| bin.488 | 79.31 | 1.006 | 0.329 | 20002 | 1830453 |
| bin.489 | 96.22 | 0.943 | 0.442 | 27079 | 1781978 |
| bin.49 | 94.01 | 5.289 | 0.53 | 12478 | 2344598 |
| bin.490 | 93.45 | 3.712 | 0.416 | 10611 | 2519953 |
| bin.491 | 95.5 | 2.247 | 0.33 | 109645 | 961975 |
| bin.492 | 77.51 | 5.155 | 0.599 | 9299 | 1906570 |
| bin.493 | 94.36 | 0.134 | 0.321 | 55003 | 2407017 |
| bin.494 | 93.75 | 1.086 | 0.293 | 53940 | 1090658 |
| bin.495 | 82.53 | 1.733 | 0.402 | 6492 | 2192415 |
| bin.496 | 91.89 | 2.237 | 0.539 | 11496 | 2280511 |
| bin.497 | 67.42 | 5.849 | 0.612 | 7737 | 1943912 |
| bin.498 | 91.03 | 3.02 | 0.575 | 9744 | 2008328 |
| bin.499 | 89.51 | 6.317 | 0.659 | 5606 | 2440569 |
| bin.5 | 53.05 | 1.724 | 0.641 | 2816 | 1105471 |
| bin.50 | 78.02 | 5.04 | 0.363 | 10634 | 913752 |
| bin.500 | 62.26 | 2.354 | 0.488 | 23460 | 1983545 |
| bin.501 | 79.05 | 3.635 | 0.625 | 11185 | 1791762 |
| bin.502 | 94.34 | 0.563 | 0.419 | 76001 | 4367505 |
| bin.503 | 68.31 | 4.494 | 0.284 | 4986 | 818100 |
| bin.504 | 51.41 | 0 | 0.334 | 5686 | 784277 |
| bin.505 | 67.16 | 0.877 | 0.276 | 11365 | 1012969 |
| bin.506 | 91.38 | 0 | 0.61 | 23922 | 1608225 |
| bin.507 | 73.2 | 5.913 | 0.649 | 8705 | 1838241 |
| bin.508 | 93.95 | 0 | 0.508 | 53751 | 2232921 |
| bin.509 | 99.24 | 2.275 | 0.499 | 97205 | 2504067 |
| bin.51 | 86.26 | 2.147 | 0.608 | 25885 | 1564125 |
| bin.510 | 51.51 | 1.754 | 0.276 | 9778 | 710939 |
| bin.511 | 87.27 | 0 | 0.289 | 15408 | 992662 |
| bin.512 | 87.29 | 6.894 | 0.376 | 9207 | 1906027 |
| bin.513 | 74.38 | 1.612 | 0.322 | 12707 | 1082712 |
| bin.514 | 88.16 | 3.063 | 0.526 | 10034 | 2506111 |
| bin.515 | 76.58 | 2.653 | 0.479 | 5980 | 2601740 |
| bin.516 | 88.89 | 3.009 | 0.606 | 15862 | 2186986 |
| bin.517 | 92.09 | 0 | 0.576 | 17145 | 1776538 |
| bin.518 | 58.75 | 4.385 | 0.276 | 9248 | 893055 |
| bin.519 | 92.72 | 5.795 | 0.384 | 10774 | 1953008 |
| bin.52 | 82.44 | 2.116 | 0.488 | 5055 | 1456753 |
| bin.520 | 85.39 | 2.344 | 0.295 | 6400 | 1042821 |
| bin.521 | 78.99 | 4 | 0.288 | 9647 | 1043165 |
| bin.522 | 94.28 | 1.793 | 0.539 | 46345 | 2908151 |
| bin.523 | 88.2 | 1.685 | 0.256 | 19613 | 989619 |
| bin.524 | 53.95 | 1.582 | 0.551 | 6930 | 1778101 |
| bin.525 | 97.31 | 0 | 0.421 | 64875 | 2016839 |
| bin.526 | 98.32 | 4.25 | 0.413 | 28941 | 2249578 |
| bin.527 | 98.52 | 8.227 | 0.501 | 13662 | 4087281 |
| bin.528 | 65.26 | 0.95 | 0.38 | 8108 | 896306 |
| bin.529 | 81.8 | 0.167 | 0.638 | 17851 | 2140473 |
| bin.53 | 88.86 | 1.785 | 0.444 | 90054 | 2189451 |
| bin.530 | 72.21 | 6.41 | 0.456 | 13454 | 829879 |
| bin.531 | 58.61 | 5.617 | 0.24 | 6196 | 731073 |
| bin.532 | 90.72 | 0.403 | 0.515 | 18130 | 1714308 |
| bin.533 | 87.31 | 3.816 | 0.448 | 14000 | 2580695 |
| bin.534 | 79.31 | 2.586 | 0.443 | 17151 | 3046264 |
| bin.535 | 91.8 | 3.02 | 0.608 | 9857 | 2087848 |
| bin.536 | 50.9 | 4.545 | 0.255 | 12010 | 1380038 |
| bin.537 | 76.77 | 7.677 | 0.285 | 4071 | 1038045 |
| bin.538 | 52.59 | 0 | 0.5 | 9091 | 1075679 |
| bin.539 | 93.76 | 1.914 | 0.518 | 18547 | 3037627 |
| bin.54 | 55.17 | 0 | 0.483 | 7158 | 2113111 |
| bin.540 | 83.18 | 4.875 | 0.505 | 5620 | 1996669 |
| bin.541 | 94.85 | 0 | 0.275 | 321630 | 1542121 |
| bin.542 | 54.31 | 3.448 | 0.456 | 4295 | 2398363 |
| bin.543 | 96.26 | 0 | 0.5 | 110589 | 2195026 |
| bin.544 | 91.66 | 0.476 | 0.508 | 53011 | 2382388 |
| bin.545 | 86.47 | 9.161 | 0.413 | 5601 | 2352842 |
| bin.546 | 68.84 | 4.362 | 0.268 | 14410 | 1396136 |
| bin.547 | 91.66 | 0.416 | 0.655 | 24638 | 1487156 |
| bin.548 | 75.41 | 6.352 | 0.65 | 5161 | 1809990 |
| bin.549 | 64.1 | 0.838 | 0.557 | 4300 | 1118019 |
| bin.55 | 80.94 | 0.943 | 0.354 | 5622 | 1657015 |
| bin.550 | 88.2 | 3.932 | 0.339 | 8191 | 1104992 |
| bin.551 | 89.82 | 0.714 | 0.548 | 18549 | 2191927 |
| bin.552 | 68.02 | 1.724 | 0.612 | 4220 | 1921164 |
| bin.553 | 71.19 | 1.461 | 0.497 | 6969 | 2363388 |
| bin.554 | 76.58 | 3.205 | 0.314 | 14129 | 1413881 |
| bin.555 | 95.94 | 2.507 | 0.464 | 13673 | 2405238 |
| bin.556 | 78.23 | 4.013 | 0.645 | 6311 | 2147191 |
| bin.557 | 67.98 | 2.122 | 0.503 | 4658 | 1992367 |
| bin.558 | 97.37 | 0.187 | 0.507 | 33330 | 2564100 |
| bin.559 | 96.27 | 7.022 | 0.296 | 12208 | 2166257 |
| bin.56 | 95.5 | 0.022 | 0.494 | 37375 | 1949739 |
| bin.560 | 85.11 | 1.123 | 0.273 | 10854 | 1116245 |
| bin.561 | 94.23 | 0.192 | 0.454 | 100841 | 4177827 |
| bin.562 | 71.59 | 3.379 | 0.245 | 23777 | 1270023 |
| bin.563 | 76.66 | 4.666 | 0.236 | 6606 | 1090750 |
| bin.564 | 64.81 | 0 | 0.278 | 28950 | 931334 |
| bin.565 | 73 | 1.7 | 0.626 | 19430 | 1595052 |
| bin.566 | 87.67 | 2.889 | 0.488 | 9027 | 1413192 |
| bin.567 | 54.14 | 2.348 | 0.268 | 9508 | 940815 |
| bin.568 | 90.44 | 1.674 | 0.505 | 8213 | 2440090 |
| bin.569 | 94.99 | 0.632 | 0.468 | 34960 | 2473019 |
| bin.57 | 51.09 | 7.482 | 0.621 | 10426 | 1691712 |
| bin.570 | 56.46 | 0 | 0.418 | 4005 | 1297444 |
| bin.571 | 68.1 | 4.753 | 0.294 | 16588 | 1236868 |
| bin.572 | 80.07 | 0.116 | 0.491 | 12781 | 1837631 |
| bin.573 | 81.68 | 9.795 | 0.484 | 4826 | 2610781 |
| bin.574 | 84.35 | 2.176 | 0.529 | 9249 | 2261205 |
| bin.575 | 84.96 | 2.715 | 0.471 | 16009 | 1843638 |
| bin.576 | 71.71 | 2.46 | 0.289 | 15622 | 1252201 |
| bin.577 | 94.89 | 2.551 | 0.591 | 20156 | 2227172 |
| bin.578 | 95.13 | 0 | 0.632 | 38483 | 2557619 |
| bin.579 | 79.92 | 1.923 | 0.623 | 14693 | 1829286 |
| bin.58 | 61.74 | 0.278 | 0.504 | 43139 | 1805995 |
| bin.580 | 68.09 | 7.046 | 0.584 | 4742 | 1808912 |
| bin.581 | 56.4 | 0 | 0.545 | 4142 | 1097175 |
| bin.582 | 93.29 | 3.897 | 0.635 | 30709 | 2783471 |
| bin.583 | 82.12 | 3.378 | 0.576 | 7826 | 1758077 |
| bin.584 | 64.8 | 3.968 | 0.497 | 6905 | 1225743 |
| bin.585 | 85.89 | 0.316 | 0.32 | 21086 | 1889591 |
| bin.586 | 91.54 | 2.926 | 0.341 | 39957 | 1878830 |
| bin.587 | 80.27 | 1.658 | 0.247 | 9951 | 865956 |
| bin.588 | 65.67 | 0.699 | 0.349 | 15932 | 1144902 |
| bin.589 | 68.04 | 9.233 | 0.493 | 3900 | 2187544 |
| bin.59 | 79.63 | 2.261 | 0.54 | 13279 | 1471025 |
| bin.590 | 70.33 | 2.247 | 0.297 | 4769 | 992010 |
| bin.591 | 88.7 | 1.464 | 0.645 | 16190 | 1889192 |
| bin.592 | 68.88 | 0.111 | 0.423 | 3616 | 1286976 |
| bin.593 | 92.14 | 0.244 | 0.386 | 16585 | 2079345 |
| bin.594 | 65.96 | 1.115 | 0.62 | 14129 | 1580305 |
| bin.595 | 87.75 | 3.206 | 0.635 | 12092 | 2086067 |
| bin.596 | 74.13 | 1.724 | 0.477 | 7706 | 1660672 |
| bin.597 | 61.46 | 4.494 | 0.314 | 3810 | 681723 |
| bin.598 | 53.4 | 5.172 | 0.615 | 7601 | 2770760 |
| bin.599 | 91.94 | 3.618 | 0.57 | 28146 | 3148009 |
| bin.6 | 69.82 | 5.257 | 0.596 | 7978 | 1966325 |
| bin.60 | 87.66 | 0.754 | 0.477 | 14088 | 2189272 |
| bin.600 | 60.14 | 0.877 | 0.429 | 4934 | 1028669 |
| bin.601 | 75.43 | 1.754 | 0.311 | 6457 | 1165672 |
| bin.602 | 67.96 | 5.323 | 0.315 | 5475 | 1712911 |
| bin.603 | 96.29 | 2.717 | 0.407 | 14003 | 1652157 |
| bin.604 | 91.45 | 1.793 | 0.47 | 12273 | 3107604 |
| bin.605 | 50.31 | 3.523 | 0.641 | 3132 | 1800452 |
| bin.606 | 95.84 | 0.158 | 0.501 | 43810 | 2402430 |
| bin.607 | 92.83 | 5.704 | 0.579 | 12288 | 2330115 |
| bin.608 | 85.79 | 2.684 | 0.505 | 7809 | 2770843 |
| bin.609 | 94.46 | 0.685 | 0.486 | 36139 | 2466988 |
| bin.61 | 51.28 | 0.382 | 0.555 | 22390 | 1110997 |
| bin.610 | 72.37 | 3.678 | 0.325 | 7133 | 1257296 |
| bin.611 | 60.62 | 2.908 | 0.281 | 18416 | 1030883 |
| bin.612 | 97.53 | 0.566 | 0.523 | 43123 | 2381594 |
| bin.613 | 51.41 | 1.902 | 0.557 | 7916 | 1697430 |
| bin.614 | 81.46 | 3.37 | 0.292 | 10256 | 1090120 |
| bin.615 | 84.79 | 0 | 0.474 | 29314 | 2846122 |
| bin.616 | 77.06 | 6.896 | 0.531 | 48447 | 2433911 |
| bin.617 | 93.6 | 2.372 | 0.421 | 10916 | 1743889 |
| bin.618 | 99.05 | 1.482 | 0.43 | 13339 | 2564193 |
| bin.619 | 93.67 | 0.714 | 0.498 | 32472 | 2257717 |
| bin.62 | 55.71 | 0.952 | 0.49 | 23336 | 1152156 |
| bin.620 | 62.37 | 1.398 | 0.307 | 6275 | 871967 |
| bin.621 | 85.24 | 1.65 | 0.531 | 19530 | 1806849 |
| bin.622 | 64.46 | 1.006 | 0.567 | 11918 | 1454101 |
| bin.623 | 92.05 | 2.873 | 0.546 | 7499 | 2114663 |
| bin.624 | 77.57 | 1.414 | 0.576 | 88142 | 2023705 |
| bin.625 | 90.73 | 0.268 | 0.481 | 16561 | 2856438 |
| bin.626 | 85.87 | 1.438 | 0.536 | 11909 | 2645506 |
| bin.627 | 92.69 | 5.056 | 0.319 | 123266 | 1358489 |
| bin.628 | 90.82 | 0.167 | 0.547 | 15452 | 1648459 |
| bin.629 | 95.4 | 5.519 | 0.656 | 15624 | 2786834 |
| bin.63 | 66.73 | 8.333 | 0.47 | 3336 | 1759721 |
| bin.630 | 94.12 | 0.624 | 0.619 | 59666 | 3451171 |
| bin.631 | 89.27 | 2.849 | 0.654 | 24318 | 2625094 |
| bin.632 | 89.89 | 0.746 | 0.473 | 78801 | 3174852 |
| bin.633 | 72.75 | 2.796 | 0.259 | 16970 | 1521704 |
| bin.634 | 92.98 | 2.668 | 0.454 | 16381 | 2194795 |
| bin.635 | 83.62 | 6.126 | 0.514 | 16500 | 2193587 |
| bin.636 | 90.75 | 2.551 | 0.462 | 46045 | 3246428 |
| bin.637 | 94.14 | 3.158 | 0.597 | 17481 | 2603077 |
| bin.638 | 80.74 | 5.617 | 0.313 | 6083 | 1012667 |
| bin.639 | 79.98 | 2.238 | 0.595 | 15814 | 1467340 |
| bin.64 | 72.85 | 1.638 | 0.567 | 21093 | 1551283 |
| bin.640 | 91.51 | 4.996 | 0.64 | 7903 | 2349155 |
| bin.641 | 89.4 | 1.027 | 0.553 | 14923 | 1595636 |
| bin.642 | 87.07 | 1.123 | 0.523 | 9086 | 1217178 |
| bin.643 | 70.25 | 2.163 | 0.569 | 4867 | 1099420 |
| bin.644 | 94.29 | 2.695 | 0.416 | 23255 | 1540436 |
| bin.645 | 86.7 | 0.709 | 0.544 | 8080 | 2221779 |
| bin.646 | 55.7 | 0.476 | 0.385 | 3168 | 903026 |
| bin.647 | 83.94 | 2.004 | 0.471 | 11003 | 2103461 |
| bin.648 | 97.71 | 0.323 | 0.417 | 101523 | 5774385 |
| bin.649 | 82.25 | 6.37 | 0.485 | 7986 | 1978048 |
| bin.65 | 60.97 | 0.134 | 0.559 | 3565 | 1215845 |
| bin.650 | 93.14 | 1.37 | 0.536 | 19505 | 1342016 |
| bin.651 | 91.32 | 3.064 | 0.603 | 16965 | 2042770 |
| bin.652 | 75.76 | 0.862 | 0.555 | 7665 | 1763280 |
| bin.653 | 86.09 | 2.21 | 0.594 | 16279 | 2047805 |
| bin.654 | 75.34 | 1.677 | 0.255 | 31975 | 1174517 |
| bin.655 | 94.29 | 3.131 | 0.392 | 14948 | 1999133 |
| bin.656 | 88.59 | 0 | 0.338 | 22569 | 2888727 |
| bin.657 | 100 | 1.509 | 0.386 | 145401 | 3167019 |
| bin.658 | 81.15 | 7.053 | 0.631 | 5430 | 1835100 |
| bin.659 | 82.62 | 2.564 | 0.32 | 13981 | 1878422 |
| bin.66 | 54.2 | 1.677 | 0.289 | 5094 | 897552 |
| bin.660 | 91.13 | 2.848 | 0.482 | 30297 | 2724819 |
| bin.661 | 91.35 | 4.382 | 0.588 | 6470 | 2470461 |
| bin.662 | 76.53 | 4.079 | 0.627 | 7829 | 2045422 |
| bin.663 | 93.17 | 3.396 | 0.522 | 33205 | 2627575 |
| bin.664 | 52.32 | 5.172 | 0.575 | 3843 | 1679454 |
| bin.665 | 93.19 | 3.656 | 0.534 | 19014 | 3279862 |
| bin.666 | 74.08 | 2.857 | 0.354 | 11139 | 1358061 |
| bin.667 | 87.46 | 1.612 | 0.528 | 10575 | 1871596 |
| bin.668 | 88.53 | 4.233 | 0.594 | 10834 | 3192434 |
| bin.669 | 70.15 | 3.382 | 0.506 | 8803 | 1774465 |
| bin.67 | 94.7 | 0.914 | 0.405 | 62479 | 3296277 |
| bin.670 | 71.18 | 0.197 | 0.527 | 6008 | 1441171 |
| bin.671 | 54.57 | 6.034 | 0.678 | 3973 | 1354060 |
| bin.672 | 62.55 | 0 | 0.405 | 16353 | 846163 |
| bin.673 | 60.86 | 4.137 | 0.626 | 6797 | 2049546 |
| bin.674 | 93.15 | 0 | 0.593 | 57913 | 2226766 |
| bin.675 | 81.56 | 1.29 | 0.431 | 19247 | 1230216 |
| bin.676 | 64.43 | 2.141 | 0.267 | 12690 | 1237366 |
| bin.677 | 99.18 | 0.581 | 0.429 | 45514 | 1772493 |
| bin.678 | 95.56 | 0.858 | 0.555 | 12943 | 1718123 |
| bin.679 | 52.93 | 0 | 0.452 | 6070 | 1089374 |
| bin.68 | 98.12 | 0.749 | 0.404 | 45886 | 1605421 |
| bin.680 | 95.97 | 0 | 0.538 | 32136 | 1700640 |
| bin.681 | 87.24 | 7.496 | 0.658 | 7306 | 2107758 |
| bin.682 | 91.5 | 1.36 | 0.649 | 16235 | 2298725 |
| bin.683 | 82.47 | 1.498 | 0.297 | 37355 | 1092821 |
| bin.684 | 67.03 | 1.724 | 0.61 | 4412 | 1492175 |
| bin.685 | 83.15 | 2.446 | 0.617 | 10165 | 2281853 |
| bin.686 | 61.3 | 2.666 | 0.263 | 19124 | 987651 |
| bin.687 | 96.55 | 2.586 | 0.47 | 115121 | 2728633 |
| bin.688 | 98.43 | 0.671 | 0.493 | 38387 | 2320859 |
| bin.689 | 76.92 | 4.029 | 0.419 | 11842 | 786836 |
| bin.69 | 51.9 | 5.172 | 0.393 | 2682 | 2116998 |
| bin.690 | 93.57 | 0.044 | 0.379 | 11890 | 1010072 |
| bin.691 | 78.52 | 5.645 | 0.656 | 10818 | 2350242 |
| bin.692 | 90.09 | 1.549 | 0.553 | 30745 | 2012242 |
| bin.693 | 95.08 | 4.138 | 0.506 | 19448 | 2272382 |
| bin.694 | 88 | 4.203 | 0.31 | 6858 | 1457139 |
| bin.695 | 64.48 | 3.66 | 0.531 | 14918 | 2101927 |
| bin.696 | 89.32 | 5.056 | 0.302 | 25019 | 1251950 |
| bin.697 | 64.65 | 1.724 | 0.453 | 13189 | 2244946 |
| bin.698 | 89.08 | 3.763 | 0.542 | 6990 | 1408485 |
| bin.699 | 85.09 | 3.296 | 0.379 | 48709 | 761257 |
| bin.7 | 95.56 | 1.008 | 0.513 | 20425 | 1240204 |
| bin.70 | 87.53 | 5.379 | 0.519 | 10667 | 2575534 |
| bin.700 | 71.33 | 6.034 | 0.317 | 10811 | 998128 |
| bin.701 | 95.01 | 2.987 | 0.323 | 14863 | 2379432 |
| bin.702 | 82.25 | 7.424 | 0.652 | 13142 | 2066753 |
| bin.703 | 94.48 | 6.666 | 0.571 | 7798 | 2381662 |
| bin.704 | 58.95 | 0 | 0.533 | 4875 | 1343368 |
| bin.705 | 82.06 | 9.874 | 0.471 | 8390 | 2203494 |
| bin.706 | 87.27 | 1.834 | 0.488 | 7033 | 2323901 |
| bin.707 | 98.27 | 8.495 | 0.516 | 157870 | 4523722 |
| bin.708 | 90.03 | 0.537 | 0.454 | 45141 | 2377531 |
| bin.709 | 94.29 | 0.223 | 0.518 | 11261 | 1771550 |
| bin.71 | 82.72 | 5.411 | 0.58 | 7209 | 2001309 |
| bin.710 | 84.59 | 8.501 | 0.626 | 6538 | 1886869 |
| bin.711 | 60.04 | 1.677 | 0.276 | 11044 | 917336 |
| bin.712 | 56.89 | 0 | 0.554 | 57151 | 1596712 |
| bin.713 | 92.62 | 2.879 | 0.325 | 12387 | 1524966 |
| bin.714 | 78.92 | 0 | 0.349 | 26106 | 1133046 |
| bin.715 | 55.5 | 3.448 | 0.403 | 9000 | 1750458 |
| bin.716 | 67.93 | 2.586 | 0.472 | 11507 | 2318504 |
| bin.717 | 90.29 | 2.5 | 0.484 | 15698 | 1666667 |
| bin.718 | 82.46 | 0 | 0.296 | 15396 | 1264395 |
| bin.719 | 61.08 | 0.671 | 0.545 | 7812 | 1335777 |
| bin.72 | 71.08 | 4.153 | 0.665 | 6140 | 2045249 |
| bin.720 | 50.57 | 3.448 | 0.625 | 5085 | 1576865 |
| bin.721 | 89.46 | 0 | 0.501 | 258993 | 1609867 |
| bin.722 | 75.75 | 3.095 | 0.586 | 10946 | 1705341 |
| bin.723 | 81.08 | 2.478 | 0.324 | 12916 | 1654562 |
| bin.724 | 76.3 | 5.216 | 0.294 | 7498 | 1122081 |
| bin.725 | 77.28 | 1.754 | 0.525 | 15549 | 3486943 |
| bin.726 | 80.02 | 6.693 | 0.439 | 16217 | 841683 |
| bin.727 | 85.41 | 2.724 | 0.6 | 65724 | 2270564 |
| bin.728 | 96.62 | 1.898 | 0.527 | 17820 | 2729537 |
| bin.729 | 75.34 | 2.848 | 0.595 | 3550 | 1468806 |
| bin.73 | 81.31 | 1.949 | 0.489 | 9804 | 2468668 |
| bin.730 | 65.42 | 4.753 | 0.585 | 3599 | 1126871 |
| bin.731 | 92.77 | 3.216 | 0.501 | 16549 | 2288951 |
| bin.732 | 91.88 | 1.428 | 0.524 | 25990 | 2350153 |
| bin.733 | 54.96 | 0 | 0.632 | 15953 | 1815213 |
| bin.734 | 66.83 | 1.685 | 0.335 | 3808 | 819074 |
| bin.735 | 51.65 | 7.211 | 0.62 | 20169 | 1726721 |
| bin.736 | 89.46 | 0.949 | 0.428 | 14289 | 2444739 |
| bin.737 | 60.92 | 1.418 | 0.583 | 5104 | 965759 |
| bin.738 | 81.81 | 3.059 | 0.498 | 5175 | 2258221 |
| bin.739 | 81.83 | 2.046 | 0.49 | 12275 | 2719385 |
| bin.74 | 85.15 | 1.898 | 0.509 | 15713 | 2324367 |
| bin.740 | 70.15 | 2.806 | 0.434 | 4924 | 1552142 |
| bin.741 | 88.02 | 2.097 | 0.509 | 12329 | 2200063 |
| bin.742 | 64.8 | 0 | 0.282 | 9455 | 1234644 |
| bin.743 | 72.81 | 3.355 | 0.634 | 5094 | 2110308 |
| bin.744 | 96.77 | 0.063 | 0.471 | 24267 | 1730786 |
| bin.745 | 59.67 | 3.784 | 0.564 | 4235 | 1183300 |
| bin.746 | 74.23 | 3.62 | 0.321 | 3537 | 2233695 |
| bin.747 | 93.68 | 2.013 | 0.413 | 11711 | 2227119 |
| bin.748 | 85.37 | 1.969 | 0.545 | 11488 | 1595929 |
| bin.749 | 76.43 | 0.932 | 0.278 | 26671 | 1081299 |
| bin.75 | 66.8 | 7.315 | 0.637 | 3184 | 1570268 |
| bin.750 | 55.64 | 3.448 | 0.624 | 3406 | 1535196 |
| bin.751 | 73.34 | 5.059 | 0.406 | 60076 | 1580068 |
| bin.752 | 59.95 | 0 | 0.288 | 5977 | 735430 |
| bin.753 | 84.39 | 2.407 | 0.268 | 17919 | 927195 |
| bin.754 | 55.52 | 7.073 | 0.287 | 4613 | 959696 |
| bin.755 | 92.61 | 0.671 | 0.322 | 20913 | 1886118 |
| bin.756 | 85.17 | 1.411 | 0.457 | 25624 | 1163588 |
| bin.757 | 62.3 | 0.671 | 0.497 | 37473 | 1198902 |
| bin.758 | 81.08 | 9.309 | 0.621 | 4100 | 2042316 |
| bin.759 | 95.23 | 1.169 | 0.514 | 40698 | 2488887 |
| bin.76 | 50.9 | 3.636 | 0.279 | 5313 | 1209164 |
| bin.760 | 89.8 | 2.673 | 0.612 | 7368 | 2752353 |
| bin.761 | 85.67 | 1.724 | 0.426 | 135495 | 5884429 |
| bin.762 | 53.44 | 1.724 | 0.641 | 4241 | 1287301 |
| bin.763 | 79.95 | 3.507 | 0.365 | 6313 | 1470228 |
| bin.764 | 67.5 | 1.436 | 0.584 | 4408 | 2037957 |
| bin.765 | 96.02 | 0.671 | 0.504 | 40156 | 2320891 |
| bin.766 | 73.01 | 1.646 | 0.464 | 8829 | 1797021 |
| bin.767 | 59.71 | 1.544 | 0.468 | 7233 | 1665830 |
| bin.768 | 88.65 | 1.955 | 0.586 | 21145 | 2235351 |
| bin.769 | 95.75 | 0 | 0.6 | 112129 | 3120686 |
| bin.77 | 63.96 | 0 | 0.472 | 6039 | 1396179 |
| bin.770 | 98.81 | 0.591 | 0.614 | 103071 | 2921460 |
| bin.771 | 95.48 | 3.184 | 0.494 | 17665 | 2846023 |
| bin.772 | 94.63 | 0.167 | 0.493 | 33786 | 2117794 |
| bin.773 | 83.28 | 9.494 | 0.661 | 4819 | 1755528 |
| bin.774 | 61.04 | 1.316 | 0.532 | 6911 | 1755203 |
| bin.775 | 97.41 | 2.329 | 0.528 | 19683 | 2472256 |
| bin.776 | 70.31 | 6.926 | 0.61 | 6086 | 1881229 |
| bin.777 | 89.48 | 2.023 | 0.499 | 16792 | 2312800 |
| bin.778 | 93.82 | 4.73 | 0.664 | 10621 | 2468699 |
| bin.779 | 91.41 | 4.72 | 0.611 | 22616 | 2257082 |
| bin.78 | 92.43 | 4.681 | 0.491 | 12423 | 1459942 |
| bin.780 | 66.29 | 1.23 | 0.636 | 15962 | 1578418 |
| bin.781 | 73.78 | 7.885 | 0.618 | 5288 | 1853185 |
| bin.782 | 92.32 | 3.138 | 0.636 | 15776 | 2172350 |
| bin.783 | 93.53 | 0.586 | 0.529 | 15124 | 3142427 |
| bin.784 | 75.32 | 2.808 | 0.267 | 21274 | 872732 |
| bin.785 | 92.58 | 3.932 | 0.263 | 29316 | 1396649 |
| bin.786 | 96.03 | 2.762 | 0.444 | 16329 | 2064162 |
| bin.787 | 51.36 | 4.31 | 0.58 | 5651 | 1836309 |
| bin.788 | 88.53 | 1.898 | 0.499 | 10328 | 2365387 |
| bin.789 | 83.17 | 3.494 | 0.652 | 17488 | 2322197 |
| bin.79 | 95.88 | 1.761 | 0.465 | 20038 | 2499375 |
| bin.790 | 78.05 | 5.333 | 0.295 | 19830 | 808811 |
| bin.791 | 73.65 | 4.194 | 0.621 | 9814 | 1924669 |
| bin.792 | 94.46 | 1.23 | 0.485 | 13925 | 2335818 |
| bin.793 | 79.9 | 2.404 | 0.582 | 7523 | 1626553 |
| bin.794 | 62.31 | 1.881 | 0.658 | 5689 | 1204548 |
| bin.795 | 98.97 | 0.471 | 0.346 | 23590 | 2437518 |
| bin.796 | 72.88 | 5.172 | 0.592 | 7197 | 1945763 |
| bin.797 | 89.73 | 3.041 | 0.612 | 21637 | 2208991 |
| bin.798 | 100 | 3.561 | 0.648 | 67526 | 2886334 |
| bin.799 | 89.81 | 2.452 | 0.455 | 16534 | 2104283 |
| bin.8 | 87.82 | 6.69 | 0.41 | 5883 | 1239263 |
| bin.80 | 88.86 | 0.632 | 0.499 | 13547 | 3080869 |
| bin.800 | 68.99 | 6.935 | 0.614 | 10922 | 1649429 |
| bin.801 | 94.38 | 0 | 0.558 | 31361 | 2210927 |
| bin.802 | 83.59 | 6.79 | 0.251 | 6638 | 1018141 |
| bin.803 | 97.86 | 0 | 0.464 | 111607 | 2211803 |
| bin.804 | 61.76 | 4.492 | 0.509 | 6425 | 2131664 |
| bin.805 | 50.9 | 5.454 | 0.252 | 7957 | 996302 |
| bin.806 | 55.86 | 3.448 | 0.512 | 6038 | 2455695 |
| bin.807 | 76.18 | 1.123 | 0.348 | 4392 | 873326 |
| bin.808 | 97.31 | 2.715 | 0.314 | 41481 | 2484346 |
| bin.809 | 50.15 | 1.724 | 0.515 | 9325 | 1291839 |
| bin.81 | 68.5 | 2.556 | 0.366 | 8788 | 866658 |
| bin.810 | 51.43 | 3.448 | 0.509 | 4999 | 1492369 |
| bin.811 | 67.65 | 1.063 | 0.344 | 17693 | 947600 |
| bin.812 | 88.88 | 1.804 | 0.334 | 132502 | 1808660 |
| bin.813 | 85.88 | 0.476 | 0.457 | 37579 | 1564522 |
| bin.814 | 92.22 | 0 | 0.573 | 73405 | 1740682 |
| bin.815 | 78.58 | 1.738 | 0.339 | 4536 | 1763536 |
| bin.816 | 86.96 | 2.948 | 0.52 | 23857 | 2473936 |
| bin.817 | 99.31 | 3.85 | 0.392 | 54471 | 3403309 |
| bin.818 | 97.78 | 3.481 | 0.49 | 15675 | 2267748 |
| bin.819 | 97.69 | 4.169 | 0.528 | 94663 | 2519993 |
| bin.82 | 94.34 | 6.771 | 0.512 | 11393 | 1240403 |
| bin.820 | 63.75 | 2.684 | 0.448 | 5430 | 1512627 |
| bin.821 | 59.99 | 1.112 | 0.554 | 5895 | 1911448 |
| bin.822 | 59.55 | 7.865 | 0.504 | 3379 | 916355 |
| bin.823 | 93.53 | 0.634 | 0.478 | 26268 | 1307165 |
| bin.824 | 96.28 | 1.901 | 0.494 | 33212 | 2723020 |
| bin.825 | 80.84 | 0.894 | 0.581 | 7095 | 1550878 |
| bin.826 | 59.43 | 0 | 0.554 | 6554 | 1365930 |
| bin.827 | 59.35 | 1.342 | 0.501 | 12588 | 1217200 |
| bin.828 | 50.21 | 5.602 | 0.518 | 9010 | 1283873 |
| bin.829 | 61.43 | 1.16 | 0.45 | 15555 | 985404 |
| bin.83 | 85.01 | 1.075 | 0.614 | 7493 | 1980750 |
| bin.830 | 77.61 | 2.276 | 0.608 | 5842 | 1391686 |
| bin.831 | 95.63 | 3.752 | 0.605 | 18535 | 1920329 |
| bin.832 | 71.03 | 0 | 0.604 | 10389 | 2166516 |
| bin.833 | 83.81 | 4.543 | 0.267 | 8939 | 945240 |
| bin.834 | 90.65 | 2.705 | 0.619 | 8045 | 1734475 |
| bin.835 | 95.97 | 2.908 | 0.429 | 12390 | 1718693 |
| bin.836 | 77.92 | 6.586 | 0.599 | 5293 | 1898099 |
| bin.837 | 72.05 | 0 | 0.453 | 27081 | 1207920 |
| bin.838 | 74.91 | 0.877 | 0.303 | 9089 | 1054585 |
| bin.839 | 83.15 | 2.607 | 0.649 | 9714 | 2088703 |
| bin.84 | 71.23 | 6.179 | 0.372 | 4311 | 781066 |
| bin.840 | 62.05 | 0.159 | 0.647 | 9056 | 2496035 |
| bin.841 | 65.67 | 1.048 | 0.683 | 17826 | 1168252 |
| bin.842 | 88.2 | 1.235 | 0.255 | 20181 | 1105428 |
| bin.843 | 68.73 | 2.327 | 0.439 | 11876 | 686818 |
| bin.844 | 98.59 | 0.117 | 0.38 | 87170 | 1597487 |
| bin.845 | 55.71 | 2.678 | 0.641 | 4423 | 1512249 |
| bin.846 | 85.46 | 5.587 | 0.587 | 9147 | 1424418 |
| bin.847 | 64.53 | 0.335 | 0.617 | 11537 | 1784957 |
| bin.848 | 98.49 | 0.754 | 0.498 | 73998 | 2738899 |
| bin.849 | 95.6 | 2.621 | 0.492 | 40647 | 3123680 |
| bin.85 | 83.35 | 4.435 | 0.626 | 4512 | 1636090 |
| bin.850 | 85.28 | 3.183 | 0.338 | 8457 | 953489 |
| bin.851 | 96.59 | 3.409 | 0.458 | 64685 | 2523475 |
| bin.852 | 55.48 | 1.724 | 0.634 | 8389 | 1561573 |
| bin.853 | 82.84 | 4.905 | 0.523 | 4836 | 1392817 |
| bin.854 | 65.46 | 0.595 | 0.551 | 10877 | 1313571 |
| bin.855 | 74.35 | 7.415 | 0.279 | 5757 | 1093264 |
| bin.856 | 52.02 | 3.987 | 0.281 | 4865 | 1280919 |
| bin.857 | 67.55 | 3.859 | 0.656 | 4061 | 1525173 |
| bin.858 | 65.79 | 1.23 | 0.55 | 14230 | 1166323 |
| bin.859 | 84.8 | 2.564 | 0.344 | 19421 | 2194643 |
| bin.86 | 95.43 | 0.949 | 0.452 | 44564 | 2706131 |
| bin.860 | 96.34 | 0.681 | 0.435 | 40608 | 3240080 |
| bin.861 | 80.4 | 1.814 | 0.57 | 9945 | 2480542 |
| bin.862 | 68.27 | 6.802 | 0.62 | 4192 | 1862147 |
| bin.863 | 91.53 | 1.086 | 0.46 | 7977 | 1369761 |
| bin.864 | 73.74 | 0.223 | 0.354 | 49268 | 1214938 |
| bin.865 | 79.51 | 7.62 | 0.518 | 5397 | 1797380 |
| bin.866 | 57.39 | 4.31 | 0.616 | 5647 | 1451518 |
| bin.867 | 87.16 | 4.326 | 0.605 | 34632 | 1485949 |
| bin.868 | 94.35 | 1.937 | 0.343 | 19273 | 1460310 |
| bin.869 | 57.78 | 2.175 | 0.331 | 3686 | 1001782 |
| bin.87 | 97.33 | 0.111 | 0.391 | 80097 | 1537621 |
| bin.870 | 93.53 | 7.763 | 0.603 | 9241 | 2076255 |
| bin.871 | 88.06 | 4.539 | 0.505 | 6129 | 1354348 |
| bin.872 | 69.95 | 0.403 | 0.641 | 3909 | 1361263 |
| bin.873 | 83.26 | 6.048 | 0.524 | 7307 | 1630323 |
| bin.874 | 78.74 | 0 | 0.654 | 10907 | 2365278 |
| bin.875 | 81.6 | 0.188 | 0.451 | 5225 | 2055520 |
| bin.876 | 65.62 | 2.516 | 0.574 | 3506 | 1591306 |
| bin.877 | 71.13 | 1.272 | 0.289 | 15317 | 1176220 |
| bin.878 | 92.33 | 0.858 | 0.453 | 54675 | 1623724 |
| bin.879 | 77.74 | 3.587 | 0.566 | 28366 | 1949945 |
| bin.88 | 97.03 | 0.37 | 0.562 | 56626 | 3484399 |
| bin.880 | 71.16 | 5.299 | 0.303 | 3565 | 1801379 |
| bin.881 | 97.25 | 2.075 | 0.471 | 20479 | 2649352 |
| bin.882 | 67.63 | 1.818 | 0.633 | 9674 | 1972080 |
| bin.883 | 50.87 | 3.508 | 0.281 | 4145 | 1046641 |
| bin.884 | 57.16 | 2.469 | 0.294 | 6074 | 1123946 |
| bin.885 | 88.47 | 7.914 | 0.297 | 15822 | 977122 |
| bin.886 | 92.42 | 1.238 | 0.449 | 12539 | 2850736 |
| bin.887 | 55.43 | 0.877 | 0.298 | 11876 | 922528 |
| bin.888 | 90.02 | 2.848 | 0.437 | 10275 | 1491353 |
| bin.889 | 60.01 | 3.448 | 0.474 | 14017 | 2042852 |
| bin.89 | 71 | 1.685 | 0.31 | 5129 | 997113 |
| bin.890 | 85.04 | 8.988 | 0.288 | 13369 | 1124245 |
| bin.891 | 89.56 | 6.519 | 0.4 | 9865 | 2406034 |
| bin.892 | 98.87 | 0.112 | 0.298 | 21794 | 1572952 |
| bin.893 | 56.81 | 0.899 | 0.601 | 7333 | 1426726 |
| bin.894 | 94.06 | 1.163 | 0.595 | 11360 | 2472367 |
| bin.895 | 85.08 | 0.403 | 0.575 | 9024 | 1927206 |
| bin.896 | 80.99 | 3.091 | 0.553 | 5341 | 1260784 |
| bin.897 | 65.4 | 6.034 | 0.369 | 5515 | 1476315 |
| bin.898 | 83.99 | 3.518 | 0.536 | 75998 | 2253545 |
| bin.899 | 79.56 | 1.351 | 0.419 | 39978 | 2099713 |
| bin.9 | 61.66 | 5.872 | 0.289 | 6642 | 1211501 |
| bin.90 | 82.52 | 0.02 | 0.547 | 47358 | 1757024 |
| bin.900 | 68.96 | 3.448 | 0.272 | 5989 | 1212453 |
| bin.901 | 93.65 | 0.549 | 0.547 | 13913 | 1783015 |
| bin.902 | 99.51 | 1.159 | 0.31 | 53382 | 1594471 |
| bin.903 | 85.95 | 8.273 | 0.532 | 4559 | 1330355 |
| bin.904 | 87.88 | 6.17 | 0.526 | 8456 | 2447648 |
| bin.905 | 88.72 | 1.115 | 0.425 | 22830 | 2473801 |
| bin.906 | 79.5 | 2.016 | 0.532 | 6030 | 1064536 |
| bin.907 | 97.63 | 0.354 | 0.471 | 23883 | 1871983 |
| bin.908 | 82.08 | 4.502 | 0.504 | 10512 | 1989158 |
| bin.909 | 78.32 | 1.677 | 0.618 | 10454 | 1839018 |
| bin.91 | 60.38 | 1.789 | 0.288 | 5815 | 894462 |
| bin.910 | 99.5 | 0.358 | 0.419 | 271658 | 2107298 |
| bin.911 | 60.71 | 5.263 | 0.262 | 5654 | 1153240 |
| bin.912 | 94.52 | 1.582 | 0.515 | 19528 | 2979199 |
| bin.913 | 97.6 | 3.696 | 0.526 | 14830 | 2861997 |
| bin.914 | 58.12 | 0 | 0.541 | 4501 | 1359532 |
| bin.915 | 95.67 | 2.478 | 0.521 | 41273 | 3702649 |
| bin.916 | 83.16 | 2.531 | 0.53 | 4415 | 1954841 |
| bin.917 | 72.48 | 0 | 0.455 | 49321 | 1707876 |
| bin.918 | 82.29 | 5.375 | 0.633 | 6147 | 2139262 |
| bin.919 | 89.6 | 2.524 | 0.547 | 28676 | 2524489 |
| bin.92 | 80.94 | 3.698 | 0.592 | 7677 | 1447988 |
| bin.920 | 74.93 | 3.552 | 0.669 | 4127 | 2698448 |
| bin.921 | 72.26 | 0.675 | 0.497 | 103338 | 1505075 |
| bin.922 | 68.53 | 6.182 | 0.647 | 5508 | 1395619 |
| bin.923 | 79.49 | 5.746 | 0.602 | 5369 | 2033336 |
| bin.924 | 65.72 | 0.899 | 0.534 | 16348 | 1504426 |
| bin.925 | 74.15 | 3.152 | 0.254 | 10939 | 1441624 |
| bin.926 | 82.33 | 2.348 | 0.605 | 11933 | 1716859 |
| bin.927 | 63.39 | 5.357 | 0.634 | 5932 | 1646310 |
| bin.928 | 88.83 | 5.536 | 0.475 | 9734 | 2334251 |
| bin.929 | 81.85 | 4.953 | 0.629 | 7732 | 2306411 |
| bin.93 | 79.41 | 7.037 | 0.512 | 6604 | 2141665 |
| bin.930 | 72.83 | 6.619 | 0.261 | 12912 | 1494452 |
| bin.931 | 74.78 | 1.906 | 0.635 | 9178 | 1761635 |
| bin.932 | 78.11 | 1.798 | 0.615 | 7043 | 1858491 |
| bin.933 | 69.47 | 4.865 | 0.597 | 3793 | 1689935 |
| bin.934 | 73.87 | 1.654 | 0.416 | 9238 | 1787422 |
| bin.935 | 94.16 | 2.013 | 0.52 | 14158 | 2348777 |
| bin.936 | 91.52 | 0.251 | 0.614 | 21128 | 1738958 |
| bin.937 | 54.75 | 1.407 | 0.644 | 10187 | 1023361 |
| bin.938 | 90.51 | 3.981 | 0.415 | 45736 | 2833486 |
| bin.939 | 91.29 | 2.247 | 0.286 | 20794 | 1006192 |
| bin.94 | 67.94 | 2.016 | 0.572 | 7241 | 1657477 |
| bin.940 | 82.8 | 4.716 | 0.307 | 4371 | 1502194 |
| bin.941 | 87.7 | 2.419 | 0.525 | 10702 | 2223710 |
| bin.942 | 75.86 | 1.724 | 0.491 | 33994 | 1446854 |
| bin.943 | 85.02 | 0.187 | 0.63 | 6249 | 1423104 |
| bin.944 | 93.08 | 2.035 | 0.489 | 12486 | 2696489 |
| bin.945 | 57.43 | 3.636 | 0.527 | 3276 | 858718 |
| bin.946 | 89.32 | 1.894 | 0.346 | 16292 | 1141564 |
| bin.947 | 91.68 | 1.758 | 0.505 | 17131 | 1633356 |
| bin.948 | 84.83 | 0.561 | 0.242 | 15902 | 1004921 |
| bin.949 | 59.64 | 2.125 | 0.281 | 10024 | 1144421 |
| bin.95 | 95.97 | 0 | 0.513 | 48940 | 1671214 |
| bin.950 | 83.75 | 6.452 | 0.5 | 9999 | 2711933 |
| bin.951 | 61.41 | 4.347 | 0.471 | 40340 | 729752 |
| bin.952 | 68.06 | 1.363 | 0.626 | 15833 | 1255528 |
| bin.953 | 94.74 | 1.7 | 0.498 | 31048 | 2913852 |
| bin.954 | 64.12 | 9.649 | 0.293 | 6343 | 885465 |
| bin.955 | 66.21 | 3.628 | 0.609 | 13027 | 2219528 |
| bin.956 | 90.84 | 5.704 | 0.571 | 15508 | 2797566 |
| bin.957 | 79.53 | 3.932 | 0.261 | 8269 | 1134865 |
| bin.958 | 57.97 | 1.724 | 0.648 | 11520 | 2157673 |
| bin.959 | 93.89 | 0.949 | 0.529 | 25477 | 2423791 |
| bin.96 | 70.6 | 8.579 | 0.635 | 4396 | 2557578 |
| bin.960 | 57.02 | 3.838 | 0.531 | 6569 | 1624582 |
| bin.961 | 88.34 | 3.995 | 0.452 | 12158 | 2696542 |
| bin.962 | 56.3 | 1.333 | 0.256 | 7452 | 637444 |
| bin.963 | 97.98 | 4.586 | 0.483 | 26133 | 2489613 |
| bin.964 | 85.59 | 3.401 | 0.622 | 12545 | 1698268 |
| bin.965 | 57.84 | 2.631 | 0.283 | 6461 | 570163 |
| bin.966 | 82.42 | 8.631 | 0.59 | 4902 | 3200129 |
| bin.967 | 88.94 | 0 | 0.466 | 37988 | 1828937 |
| bin.968 | 78.12 | 1.346 | 0.391 | 11543 | 815013 |
| bin.969 | 66.63 | 3.467 | 0.563 | 4997 | 1563466 |
| bin.97 | 78.65 | 0.65 | 0.456 | 15191 | 2278382 |
| bin.970 | 96.64 | 0 | 0.335 | 57877 | 2273247 |
| bin.971 | 75.74 | 6.282 | 0.424 | 4703 | 2489136 |
| bin.972 | 66.23 | 1.342 | 0.261 | 27273 | 1285879 |
| bin.973 | 76.22 | 6.854 | 0.641 | 9938 | 3362503 |
| bin.974 | 90.72 | 0.481 | 0.366 | 48239 | 1463796 |
| bin.975 | 87.77 | 3.92 | 0.533 | 13090 | 2020321 |
| bin.976 | 55.75 | 0 | 0.455 | 11060 | 2632235 |
| bin.977 | 87.01 | 3.333 | 0.489 | 14637 | 2151436 |
| bin.978 | 87.69 | 5.777 | 0.619 | 5730 | 1981902 |
| bin.979 | 94.03 | 0 | 0.464 | 16415 | 1669899 |
| bin.98 | 92.54 | 6.606 | 0.347 | 5310 | 1227124 |
| bin.980 | 80.95 | 3.355 | 0.592 | 24397 | 2431646 |
| bin.981 | 80.37 | 5.872 | 0.616 | 4542 | 2015038 |
| bin.982 | 85.42 | 6.41 | 0.537 | 10683 | 2453167 |
| bin.983 | 63.3 | 0 | 0.26 | 5887 | 1215809 |
| bin.984 | 92.76 | 2.214 | 0.481 | 25899 | 1735388 |
| bin.985 | 96.07 | 0.07 | 0.492 | 42768 | 2278377 |
| bin.986 | 73.91 | 1.77 | 0.253 | 23530 | 1369479 |
| bin.987 | 88.26 | 2.32 | 0.512 | 12377 | 2480600 |
| bin.988 | 57.86 | 5.879 | 0.594 | 2819 | 1024414 |
| bin.989 | 97.58 | 0 | 0.38 | 70793 | 2404419 |
| bin.99 | 67.24 | 0 | 0.534 | 32116 | 2234747 |
| bin.990 | 93.95 | 2.013 | 0.467 | 19859 | 1770234 |
| bin.991 | 93.05 | 0.692 | 0.556 | 47834 | 2381864 |
| bin.992 | 68.64 | 3.296 | 0.251 | 10524 | 1149152 |
| bin.993 | 54.5 | 3.007 | 0.645 | 4428 | 1528933 |
| bin.994 | 84.61 | 1.196 | 0.385 | 31977 | 1638973 |
| bin.995 | 50.15 | 6.896 | 0.606 | 5872 | 1954426 |
| bin.996 | 81.63 | 0.503 | 0.479 | 10041 | 2170303 |
| bin.997 | 96.14 | 2.169 | 0.527 | 22591 | 2332303 |
| bin.998 | 71.23 | 4.952 | 0.575 | 3557 | 1219371 |
| bin.999 | 54.11 | 2.482 | 0.266 | 11297 | 1108292 |
